# Whole genome sequencing of *Moraxella bovis* strains from North America reveals two genotypes with different genetic determinants

**DOI:** 10.1186/s12866-022-02670-3

**Published:** 2022-10-21

**Authors:** Emily L. Wynn, Matthew M. Hille, John Dustin Loy, Gennie Schuller, Kristen L. Kuhn, Aaron M. Dickey, James L. Bono, Michael L. Clawson

**Affiliations:** 1 United States Department of Agriculture (USDA), Agricultural Research Service (ARS), United States Meat Animal Research Center, 844 Rd 313, Clay Center, NE 68901 USA; 2 School of Veterinary Medicine and Biomedical Sciences, University of Nebraska-Lincoln, Lincoln, NE USA

**Keywords:** Core genome, Genotype, Infectious bovine keratoconjunctivitis, *Moraxella bovoculi*, *Moraxella bovis*, Outer membrane protein

## Abstract

**Background:**

*Moraxella bovis* and *Moraxella bovoculi* both associate with infectious bovine keratoconjunctivitis (IBK), an economically significant and painful ocular disease that affects cattle worldwide. There are two genotypes of *M. bovoculi* (genotypes 1 and 2) that differ in their gene content and potential virulence factors, although neither have been experimentally shown to cause IBK. *M. bovis* is a causative IBK agent, however, not all strains carry a complete assortment of known virulence factors. The goals of this study were to determine the population structure and depth of *M. bovis* genomic diversity, and to compare core and accessory genes and predicted outer membrane protein profiles both within and between *M. bovis* and *M. bovoculi*.

**Results:**

Phylogenetic trees and bioinformatic analyses of 36 *M. bovis* chromosomes sequenced in this study and additional available chromosomes of *M. bovis* and both genotype 1 and 2 *M. bovoculi,* showed there are two genotypes (1 and 2) of *M. bovis.* The two *M. bovis* genotypes share a core of 2015 genes, with 121 and 186 genes specific to genotype 1 and 2, respectively. The two genotypes differ by their chromosome size and prophage content, encoded protein variants of the virulence factor hemolysin, and by their affiliation with different plasmids. Eight plasmid types were identified in this study, with types 1 and 6 observed in 88 and 56% of genotype 2 strains, respectively, and absent from genotype 1 strains. Only type 1 plasmids contained one or two gene copies encoding filamentous haemagglutinin-like proteins potentially involved with adhesion. A core of 1403 genes was shared between the genotype 1 and 2 strains of both *M. bovis* and *M. bovoculi*, which encoded a total of nine predicted outer membrane proteins.

**Conclusions:**

There are two genotypes of *M. bovis* that differ in both chromosome content and plasmid profiles and thus may not equally associate with IBK. Immunological reagents specifically targeting select genotypes of *M. bovis*, or all genotypes of *M. bovis* and *M. bovoculi* together could be designed from the outer membrane proteins identified in this study.

**Supplementary Information:**

The online version contains supplementary material available at 10.1186/s12866-022-02670-3.

## Introduction

Infectious bovine keratoconjunctivitis (IBK), also known as pinkeye, is the most common ocular disease that affects cattle with an estimated worldwide prevalence of 2.8% in beef cattle [1, 2]. IBK is a complex disease that is influenced by multiple factors involving primarily bacterial pathogens, the environment, and the host [1–4]. There is currently no widely accepted case definition for IBK, although one was recently proposed [5]. Clinical signs include edema, ulceration and perforation of the cornea, blepharospasm, photophobia, lacrimation, and permanent blindness in severe cases along with a herd level or population-based threshold [2, 5]. IBK is both an animal welfare and production loss concern, as it is painful and affected cattle can have reduced weight gain [6, 7]. Economic impacts have been insufficiently studied but costs in the US alone likely are in the hundreds of millions of dollars each year [1]. The only antibiotics with label approval for IBK treatment in the US are oxytetracycline and tulathromycin, which raises concerns for increased frequencies of bacteria with antimicrobial resistance (AMR) to these antibiotics in cattle [8–10].

There are multiple species of bacteria that have been implicated for having a role in IBK [11, 12]. Two in particular, *Moraxella bovis* and *Moraxella bovoculi* associate with a majority of IBK cases [11, 13]. *M. bovis* has been experimentally demonstrated to cause IBK, and has virulence factors that include a repeats in toxin (RTX) commonly referred to as hemolysin, cytolysin, or cytotoxin (MbxA), and type IV pili with pilin subunits (PilA) that are diversified through gene inversion and represent at least seven serologically distinct types [11, 14–18]. Both MbxA and PilA are likely necessary virulence factors, and hemolysin rich extract in the absence of *M. bovis* cells can cause IBK like lesions [16]. *M. bovis* can also harbor plasmid encoded filamentous haemagglutinin-like proteins that could be involved with adhesion [19]. There is a strain effect to *M. bovis* virulence and association with IBK that is not fully characterized, as some strains have MbxA and/or the plasmid encoding filamentous haemagglutinin-like proteins, and some do not [11]. Additionally, *M. bovis* can be found in both healthy and diseased eyes, making the distinction between primary and opportunistic pathogen unclear, and further study at the genomic level warranted [20, 21].

*M. bovoculi* has not been shown to cause IBK, however, it often is the only *Moraxella* species isolated from clinical samples from IBK cases [13, 22]. While the causal role(s) of *M. bovoculi* in IBK remains unclear, whole genome sequencing of a diverse collection of strains has shown that there are two major genotypes of *M. bovoculi* (1 and 2) that differ in their gene content of potential virulence determinants including hemolysin and AMR genes [10]. Representatives of both genotypes have been found in cattle eyes without IBK, whereas only genotype 1 has been identified in IBK eyes [10, 23]. This observation is partly the result of historical ascertainment biases in identification methods for *M. bovoculi*, and it remains an open question if the two genotypes associate differently with IBK [10].

Identifying the subspecies composition of *M. bovis*, its gene content, and its diversity at the population level are important components in understanding the extent of its primary or opportunistic pathogenicity and virulence in regards to IBK. Despite known strain effects of *M. bovis* virulence, there has been a paucity of sequenced and fully assembled *M. bovis* genomes in public databases for strain comparisons [24]. A goal of this study was to produce fully assembled genomes of a diverse collection of *M. bovis* strains that were isolated from cattle in North America, identify any subspecies at the genotype level, and to compare their genome content, including genes encoding PilA, MbxA, and predicted outer membrane proteins (OMPs). Additionally, we compared gene content and predicted OMPs between *M. bovis* and *M. bovoculi*. This could ultimately lead to the development of precision therapeutics or immunological agents that target *Moraxella* either across species, or at the substrain level.

## Results

### Whole genome sequencing of *M. bovis* and identification of two genotypes

**Fig. 1:**
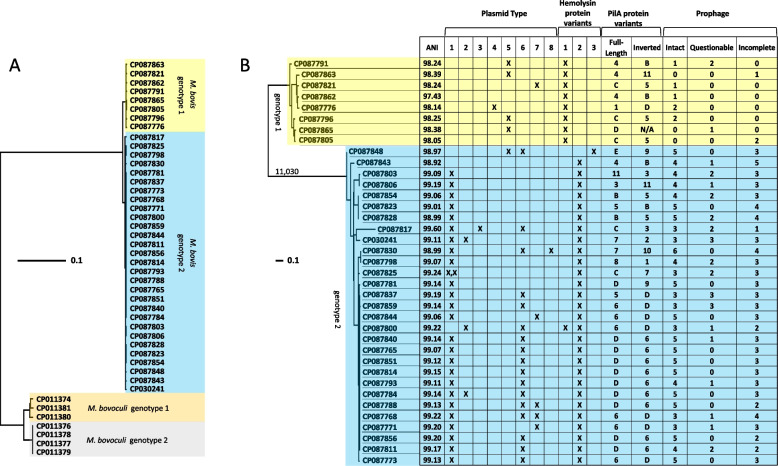
*M. bovis* genotype 1 and 2 differences. **A** Whole genome tree of *M. bovis* and *M. bovoculi* constructed in EDGAR using 1220 genes per genome, 1,278,792 bp per genome, with CP087830 as a reference. **B** Whole genome tree of *M. bovis* constructed with Parsnp using 74% of CP030241 as a reference and viewed with Ginger. The alleles of 11,070 polymorphisms separate the two genotype clades. ANI values were calculated using the whole genome sequence of *M. bovis* type strain CCUG 2133 M. Hemolysin protein variant sequences are provided in Supplementary Fig. S1. Isoform sequences of PilA are provided in Supplementary Fig. S3. PilA protein variant sequences connected to serotypes are represented with letters whereas isoforms without corresponding serotypes are represented with numbers

Thirty-six strains of *M. bovis* representing seventeen U.S. States and one Canadian province were sequenced on both PacBio and Illumina platforms and assembled into closed, circularized chromosomes (Supplementary File S1). Median and average genome coverage from the combined platforms was 535- and 536-fold respectively, with a high of 1375 and a low of 57. A Neighbor-Joining tree made in EDGAR with the 36 genomes of *M. bovis* strains sequenced in this study, and additional complete genomes of *M. bovis* (*N* = 1) and *M. bovoculi* (*N* = 7) that were available in GenBank, placed *M. bovis* and *M. bovoculi* into two well separated monophyletic clades (Fig. 1, Supplementary File S1). *M. bovoculi* further separated into two smaller clades represented by genotype 1 and 2 subtypes that have been previously described [10, 23].

The monophyletic clade of *M. bovis* also separated into two smaller clades within the Neighbor-Joining tree, which were reproduced in an Approximately Maximum-Likelihood tree produced with Parsnp (Fig. 1). A total of 32,757 polymorphisms were identified within *M. bovis*, with 11,030 supporting the separation of the two smaller *M. bovis* clades (Fig. 1, Supplementary Table S2). Average nucleotide identity (ANI) values calculated for all 37 *M. bovis* strains with completely assembled genomes, and with *M. bovis* type strain CCUG2133 as a reference, ranged from 98.14 to 99.60 (Fig. 1, Supplementary File S1), indicating that all *M. bovis* strains were properly classified at the species level, and that the two clades of *M. bovis* represented genotypes (1 and 2) at the subspecies level.

### Comparisons of plasmids between *M. bovis* genotypes 1 and 2

Eight distinct plasmid types were identified across the 36 *M. bovis* strains sequenced in this study from either PacBio assemblies or from de novo assemblies of Illumina reads that did not map to PacBio assembled genomes, as well as from *M. bovis* strain Epp63, which had previously been sequenced on PacBio and Illumina platforms (Fig. 1, Supplementary File S1). The highest frequency plasmid (type 1) was observed in 26 of 29 genotype 2 strains. This same plasmid type had previously been identified in *M. bovis* Epp63, where it contained two genes encoding filamentous haemagglutinin-like proteins potentially involved with adhesion [19]. Either one or two homologous genes encoding haemagglutinin-like proteins were observed in all type 1 plasmids identified in this study. Homologs were not observed in plasmid types two through eight.

Plasmid types 2, 3, and 4 were of low frequency in either genotype 1 or 2 strains. Plasmid type 5 was observed in four of eight genotype 1 strains and just one of the genotype 2 strains and contained toxin-antitoxin genetic machinery. Plasmid type 6 was observed in 17 of 29 genotype 2 strains and co-occurred with type 1 plasmids in 15 genotype 2 strains (Fig. 1, Supplementary File S1). All versions of type 6 plasmids encoded a zeta toxin family protein. Plasmid type 7 was observed in single representatives of both genotypes. Plasmid type eight was observed in a single genotype 2 strain and had a size of 129.6 kb.

### Comparison of hemolysin and pilin genes and proteins between *M. bovis* genotypes 1 and 2

Regarding MbxA, only three different protein variants were identified (Fig. 1, Supplementary File S1 and Supplementary Fig. S1). All eight genotype 1 *M. bovis* strains encoded the same hemolysin protein variant (variant 1). Twenty-eight of twenty-nine genotype 2 *M. bovis* strains encoded a second hemolysin protein variant (variant 2), which differed from variant 1 by eight amino acid substitutions near the C-terminus of the protein and one amino acid substitution (K171Q) located closer to the N-terminus of the protein, with genotype 2 strains having the Q allele and genotype 1 strains having the K allele. The third hemolysin protein variant was observed only in a genotype 2 *M. bovis* strain with GenBank accession number CP087848. This variant was identical to variant 2 with the exception of having the K allele at K171Q (Supplementary Fig. S1).

At the nucleotide level, a total of 28 single nucleotide polymorphisms (SNPs) were observed in *mbxA* (Supplementary Fig. S2). Excluding the genotype 2 *M. bovis* strain with GenBank accession number CP087848, the alleles of all 28 SNPs were completely linked and genotype specific. The *mbxA* of CP087848 appears to be a recombinant of genotype 1 and 2 *mbxA* haplotypes. The first three SNPs on the N-terminus side of the gene, which include two synonymous mutations and the K allele of K171Q, are genotype 1 specific alleles and the remaining 25 SNPs are all genotype 2 specific alleles (Supplementary Fig. S2). Consequently, only three unique *mbxA* haplotypes were observed in this study. A phi test for recombination conducted on the three unique *mbxA* haplotypes observed within the 37 *M. bovis* strains was inconclusive due to a paucity of informative characters.

*M. bovis pilA* and downstream sequence contain a pair of inverted repeats that can cause an inversion of the C-terminal end of *pilA* and allow a strain to express two different PilA protein variants depending on the phase of the inversion [17]. All of the genomes examined in this study except for a genotype 1 strain with GenBank accession number CP087865 encoded two different versions of *pilA* along the inversion region. CP087865 has a 4326 bp deletion downstream of the full-length *pilA* that encompasses the entirety of the region encoding the inverted version. Given that *pilA* undergoes an intrachromosomal inversion, a phi test for recombination conducted on an approximately 3200 bp chromosomal segment containing both versions of *pilA* was significant (*p* = 0.0), (Supplementary File S2). Accounting for both phases of the inversion, fifteen different protein variants of PilA were identified among *M. bovis* strains examined in this study (Supplementary Fig. S3 and Supplementary File S3). Eleven of the variants were novel to this study, while the other four corresponded with the variants of *M. bovis* strain representatives of PilA serological groups B, C, D, and E [25]. PilA serogroup reference A, F, and G sequences were not encoded by any of the genomes examined in this study.

### Comparison of clustered regularly interspaced short palindromic repeats (CRISPR) elements between *M. bovis* genotypes 1 and 2

Each of the 37 *M. bovis* genomes examined in this study contained at least one CRISPR repeat region (Supplementary File S4). All eight genotype 1 *M. bovis* strains contained three CRISPR repeat regions with different consensus repeat sequences. One genotype 1 *M. bovis* strain (GenBank accession number CP087765) contained a type 1 plasmid (CP087766) that contained an additional CRISPR repeat region. Most genotype 1 *M. bovis* strains contained a type 1-F Cas system. Genotype 1 strains with GenBank accession numbers CP087821 and CP087862 did not encode any detectable Cas genes within their genomic DNA, however CP087821 contained a type 7 plasmid (CP087822) that encoded a Cas2 gene.

All 29 genotype 2 *M. bovis* strains contained between two to five CRISPR repeat regions with different consensus repeat sequences. Twenty-three genotype 2 *M. bovis* strains contained type 1 plasmids with an additional CRISPR repeat region. Genotype 2 strain with GenBank accession number CP087817 contained an additional type 3 plasmid (CP087819) with a second additional CRISPR repeat region. Most genotype 2 *M. bovis* strains contained both a type 1-F Cas system and a type V Cas system. Four genotype 2 strains (GenBank accession numbers CP087765, CP087844, CP087768, and CP087773) did not encode any Cas genes within their genomic DNA, however CP087844 contained a type 7 plasmid (CP087846) that encoded a Cas2 gene. Full information on the CRISPR repeats and Cas genes for each strain can be found in Supplementary File S4. Seven of the genotype 1 *M. bovis* and six of the genotype 2 *M. bovis* genomes examined in this study contain CRISPR repeat regions in which CRISPR spacer regions had high sequence similarity to other regions of the genome, indicating that expression of the CRISPR system could potentially be self-detrimental.

### Comparison of prophage abundance and tandem repeats between genotypes 1 and 2

One or two prophages were detected in genotype 1 strains versus six to eleven in genotype 2 strains (Supplementary Files S1 and S5). For both genotypes, some prophages were intact and some were partial. The size of the prophages in genotype 1 strains ranged from 10.1 kb (partial) to 48.9 kb (intact). In genotype 2 strains, prophage sizes ranged from 6.2 kb (partial) to 78.5 kb (intact). The larger numbers of prophages in genotype 2 strains correspond with larger genome sizes. The average genome size of genotype 1 strains sequenced in this study was 2.58 Mb with a low of 2.54 and a high of 2.62 Mb, whereas the average for genotype 2 strains was 2.78 MB with a low of 2.68 MB and a high of 2.95 MB.

Fifteen genotype 2 strains had prophages containing one to three genes encoding TonB dependent receptors implicated in iron, or iron compound transport versus none of the genotype 1 strains (Supplementary File S5). Additionally, just one genotype 1 strain had a prophage containing toxin antitoxin system genes versus all 28 genotype 2 strains (Supplementary File S5). The differences between the genotypes indicate that genotype 2 may have gained fitness through prophages in terms of acquiring iron in the host and adapting to environmental stresses.

Both genotype 1 and 2 *M. bovis* genomes produced in this study contain multiple tandem repeats that can extend for hundreds of bases. For example, the genome of genotype 1 strain CP087791 contains a tandem repeat of six nucleotides that occurs in 55.7 copies (CP087791: 1,696,203-1,696,535), and another of 306 nucleotides that occurs in 3.7 copies (CP087791: 2,165,456-2,166,583). Genotype 2 strain CP087773 contains a tandem repeat of seven nucleotides that occurs in 48.1 copies (CP087773: 784,163-784,499) and another of 342 nucleotides that occurs in 11 copies (CP087773: 2,224,989-2,228,753). Tandem repeats in strains of both genotypes are located close to or within hypothetical genes whose function and expression profiles are currently unknown.

### Comparison of AMR genes on the chromosomes of genotype 1 and 2 *M. bovis*

A single chromosomal AMR gene encoding resistance to beta lactams including amoxicillin, ampicillin, penicillin, piperacillin, and amoxicillin was identified on six of eight genotype 1 strains, and twenty-seven of the thirty-six *M. bovis* strains sequenced in this study. The AMR gene was also not observed on the previously sequenced genotype 2 *M. bovis* strain Epp63 (Supplementary Table S2).

### Comparisons of *M. bovis* and *M. bovoculi* pan and core genomes and OMPs

**Fig. 2:**
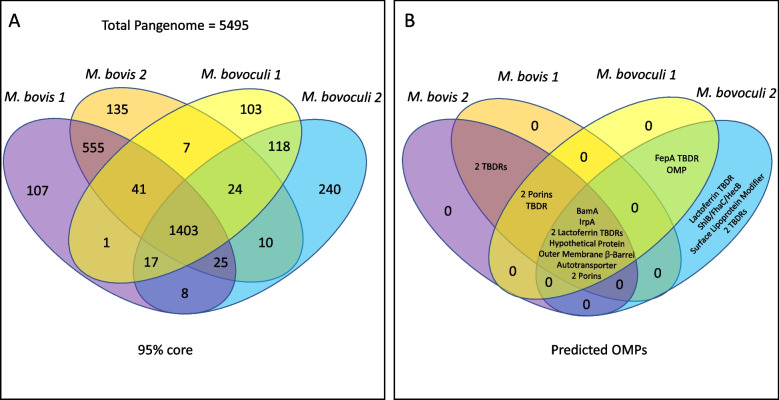
Core genome and OMP comparisons of *M. bovis* genotypes 1 and 2 and *M. bovoculi* genotypes 1 and 2. **A** Exclusive core genome of all combinations for genotypes of *M. bovis* and *M. bovoculi*. Genes represented in each slice of the Venn diagram are found in 95% of the genomes of samples from that group of genotypes and no genomes of samples from outside of that group of genotypes **B** Predicted OMPs shared between all combinations of genotypes of *M. bovis* and *M. bovoculi*

**Fig. 3:**
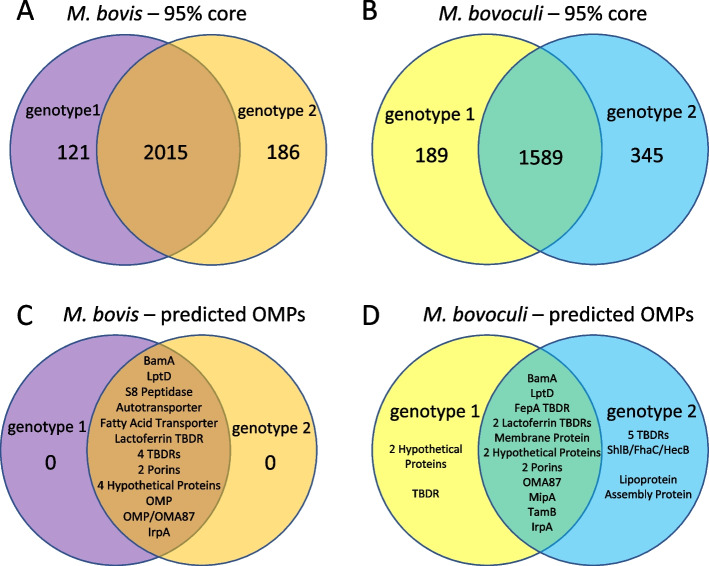
Core genome and OMP comparisons of *M. bovis* and *M. bovoculi.* **A** *M. bovis* genotypes 1 and 2 core genomes. **B** *M. bovoculi* genotypes 1 and 2 core genomes. **C** *M. bovis* genotype 1 and 2 core genome predicted OMPs. **D** *M. bovoculi* genotype 1 and 2 core genome predicted OMPs

A pan-genome of 5495 genes and a core genome of 1403 genes were identified from the 44 genomes of the two *Moraxella* species analyzed in this study (Fig. 2, Supplementary File S6). At the species level, a core genome of 2015 genes was identified for *M. bovis* with 121 genes exclusive to *M. bovis* genotype 1 and 186 exclusive to *M. bovis* genotype 2. Additionally, a core genome of 1589 genes was identified for *M. bovoculi* with 189 exclusive to *M. bovoculi* genotype 1 and 345 exclusive to *M. bovoculi* genotype 2 (Fig. 3). Within these species level core genomes, 555 genes were identified to be exclusive to *M. bovis* (not found in any *M. bovoculi* genomes), and 118 genes were identified to be exclusive to *M. bovoculi* (not found in any *M. bovis* genomes), (Fig. 2). At the genotype level of core genome comparisons, 107 genes were identified to be exclusive to genotype 1 *M. bovis*, 135 genes were identified to be exclusive to genotype 2 *M. bovis*, 103 genes were identified to be exclusive to genotype 1 *M. bovoculi*, and 240 genes were identified to be exclusive to genotype 2 *M. bovoculi* (Fig. 2).

Nine genes encoding predicted OMPs were identified in the shared core genome of all four genotypes of *M. bovis* and *M. bovoculi*: an autotransporter, a BamA protein, two porins, two TonB-dependent lactoferrin/transferrin receptors, an IrpA TonB-dependent receptor, an outer membrane beta barrel containing protein, and an uncharacterized hypothetical protein (Fig. 2). For each predicted OMP within the shared core genomes of each *Moraxella* genotype, the length of the full consensus protein, the length of the consensus predicted extracellular loop (EL) domains, and the range of sequence similarity between all genomes can be found in Supplementary File S7. Also, see Supplementary File S8 for pairwise sequence similarities of all amino acid sequences, and Supplementary File S9 for amino acid sequences of each predicted OMP.

Several predicted OMPs encoded by genes within the shared core genome of all four genotypes of *M. bovis* and *M. bovoculi* have high sequence similarity in their predicted EL domains. The consensus sequence of all TonB-dependent lactoferrin/transferrin receptor 2 protein variants is 906 amino acids long, with 462 amino acids predicted to localize to EL domains. The full length TonB-dependent lactoferrin/transferrin receptor 2 proteins have a minimum similarity of 98.7% between all genomes and the EL predicted domains have a minimum similarity of 97.4%. The consensus sequence of all outer membrane beta barrel protein variants is 200 amino acids long, with 84 amino acids predicted to localize to EL domains. The full-length outer membrane beta barrel proteins have a minimum similarity of 97.5% between all genomes and the EL predicted domains have a minimum similarity of 96.4% (Supplementary File S7).

Three genes encoding predicted OMPs were found in the shared core genome of genotype 1 *M. bovis*, genotype 2 *M. bovis*, and genotype 1 *M. bovoculi*, but not in any genomes of genotype 2 *M. bovoculi*: a TonB-dependent receptor and two porins. Of those, the TonB-dependent receptor protein variants have high sequence similarity in the predicted EL domains.

The full length TonB-dependent receptor protein variants have a minimum similarity of 93.8% between all genomes and the EL predicted domains have a minimum similarity of 97.5%. The consensus sequence of the TonB-dependent receptors is 850 amino acids long, with 432 amino acids predicted to localize to EL domains (Supplementary File S7).

## Discussion

Despite its importance with regards to pinkeye, there has been a paucity of fully assembled genomes for *M. bovis* prior to this study*. M. bovis* strains of both genotypes have long tandem repeats and repetitive phage integrations that can hinder accurate assemblies of closed bacterial genomes from short sequence platforms, as proper and accurate placement of short reads within repeat regions are difficult for the assemblers [26]. This can impact accurate detection of SNPs within repeat regions, quantitating the number of tandem repeats and prophages on a chromosome, and successfully closing a chromosome. Consequently, the longer reads produced on the PacBio platform that spanned repeat regions within the genomes assembled in this study were essential for quality control checks that the number and sequence of phage and tandem repeat motifs represented in the assemblies were accurate, and ultimately for producing closed, circular chromosomes. Given the potential importance of phenotypic variation from prophage gene activity and tandem repeat variation, the benefit of producing closed, circular *M. bovis* chromosomes for biological studies and downstream commercial applications may outweigh the speed and convenience of assembling strain genomes into multiple contigs exclusively with short read sequencing.

Plasmids are integral components of the *M. bovis* genome, perhaps more so for genotype 2 strains versus genotype 1, as 62.5% of the genotype 1 *M. bovis* strains had at least one plasmid, versus 96.5% of the genotype 2 *M. bovis* strains. High plasmid retention in genotype 2 strains may in part be due to toxins, like those of the zeta family that are encoded on plasmid type 6, that selectively kill cells that have lost the toxin encoding plasmid [27]. Differences between host plasmid profiles can be from plasmid incompatibility, where different plasmid types with isologous origins of replication or incompatible partitioning determinants can be incompatible within a cell [28]. Of the three most frequent plasmid types observed across *M. bovis* (1, 5, and 6), types 1 and 6 co-occurred across multiple genotype 2 strains. Although plasmid types 1 and 6 were specific to genotype 2 strains and plasmid type 5 was observed predominantly in genotype 1 strains, a single genotype 2 *M. bovis* did contain plasmid types 5 and 6. This indicates that all three plasmid types are compatible with each other.

There are major frequency differences of known or suspected virulence factors between the two *M. bovis* genotypes. Plasmid type 1, which was observed only in genotype 2 strains, was the only plasmid type to carry one or two copies of the haemagglutinin-like proteins that are suspected to have a role in adhesion (Fig. 1). Further, individual genotype 2 strains could have more than one type 1 plasmid (Fig. 1). Adhesins often have important roles in the attachment of pathogenic bacteria to mucus membranes, such as the conjunctiva [29], and a filamentous-haemagglutinin-like protein in particular has been shown to be critical for trachea colonization by *Bordetella bronchispetica* in a rat model [30]. Because genotype 1 strains are missing type 1 plasmids and the adhesins encoded on them, genotype 2 strains could have an advantage over genotype 1 strains in invading niches in the bovine eye. Adhesion binding variation could also occur within genotype 2 strains, which can have between zero to two type 1 plasmids, and between zero to four haemagglutinin proteins per strain, potentially affecting pathogenicity and virulence.

*M. bovis* has a four gene operon involved in the production and secretion of hemolysin along with a closely associated *tolC* gene [31, 32]. Strains with this operon can exhibit beta-hemolysis when cultured on blood agar [33, 34]. *M. bovis* strains are known to lose both their hemolysin operon genes and hemolytic activity with passage [32, 35]. All *M. bovis* strains sequenced in this study were low passage and contained complete hemolysin operons and *tolC* that were flanked on both sides by transposase genes. Each of the two *M. bovis* genotypes have distinct *mbxA* haplotypes that encode different protein variants of hemolysin. Additionally, the genotype 2 *M. bovis* strain with accession number CP087848 has an *mbxA* sequence that appears to be a linear chimera of genotype 1 and 2 *mbxA* sequences. Recombination within the hemolysin operon has been previously observed in genotype 1 *M. bovoculi* [10]. While the comparative virulence of all three hemolysin protein variants is currently unknown, all three could be collectively targeted with immunological agents in future efforts to mitigate *M. bovis* IBK cases.

Piliation is necessary for *M. bovis* pathogenicity, as strains not expressing the fimbrial protein have not been shown to cause disease [36]. Pili based vaccines have been developed to protect against IBK, and their efficacy in a challenge model has been shown to be serogroup specific [37]. Of the eleven novel variants of *M. bovis* PilA identified in this study, some were overrepresented in one genotype versus the other, such as the frequency of PilA variant 3 in the forward orientation of genotype 1 strains (37.5%) versus genotype 2 strains (3.4%), and the co-occurrence of variant 1 and serogroup D in genotype 2 strains (52%) versus genotype 1 strains (0%), (Fig. 1).

Notably, during the assembly of several *M. bovis* genomes sequenced in this study, we observed *pilA* sequence reads in both phase orientations of the inversion, indicating that populations of both *pilA* phases were present during low passage culture of the strains for sequencing, and that the frequency of phase switching can be high amongst *M. bovis* strains. Forward and inverted *pilA* sequences tested positive for recombination and show evidence of disrupted linkage between gene combinations. For example, *pilA* sequence corresponding to serogroup D and variant 6 occur together in fifteen genotype 2 strains (Fig. 1), however, serogroup D also occurs with variants 5 and 9 in genotype 2 strains and variant 1 in a genotype 1 strain. Thus, the linkage of serogroup D sequence with variant 6 has been disrupted with a concentrated gain of considerable diversity that could have come from one or more recombination event.

Gene inversion can allow *M. bovis* to undergo serogroup switching at the strain or isolate level, and two different variants of the fimbrial protein encoded by *pilA* can be expressed either simultaneously or at different time points. Due to the likelihood that recombination has occurred at the *pilA* locus between strains, it is important that 1) DNA tests to determine the molecular serogroup of an *M. bovis* strain account for *pilA* variation and can confirm the correct phase of the expressed protein, and 2) at a minimum, strains used as serogroup references are sequenced at the same time they are used as antigens for serum generation. However, given the fluidity of *pilA* inversions, it may be much more preferable to clone *pilA* into expression vectors for antigen production and subsequent serum generation, which would unambiguously connect back to *pilA* sequence and provide an unambiguous reference for a PilA protein variant sequence and serogroup.

*M. bovis* PilA diversity at the individual and population levels likely poses a challenge in its use as an effective vaccine antigen in the field. Recombinant *M. bovis* PilA vaccines are protective against the development of IBK in controlled studies using homologous strain challenge [38]. However, field trials have been less effective [39], potentially because the diversity of PilA in the field could surpass immunological protection induced by specific PilA protein variants of a recombinant or autogenous vaccine. Although multiple antigens can be incorporated into a vaccine, a diminishing level of protective antibodies can result [40, 41], highlighting a critical balance in vaccine design between adequate representation of antigen diversity in the vaccine versus protective levels of antibody induction by the host.

CRISPR-Cas systems are part of the bacterial immune system, in which CRISPR spacer regions contain DNA that can target Cas proteins to invasive genetic elements for cleavage [42]. Every *M. bovis* genome examined in this study contained CRISPR repeat regions. However, two genotype 1 *M. bovis* and four genotype 2 *M. bovis* do not encode any Cas proteins within their genomes. Two of these strains contain plasmids that encode a Cas2 gene, however they apparently still lack a fully functional Cas system. These strains that lack a functional Cas system may be more susceptible to genetic invasion than strains which contain functional CRISPR-Cas systems.

Seven of the genotype 1 *M. bovis* and six of the genotype 2 *M. bovis* strains examined in this study contain CRISPR spacer regions containing DNA that may be capable of targeting sites within the *M. bovis* genome. If properly expressed and targeted, such CRISPR self-targeting can be lethal for a bacterial cell. Due to this lethality, many species have evolved anti-CRISPR genes (*Acr*) to suppress the function of self-targeting CRISPRs. Previously, several novel *Acrs* were identified in *M. bovoculi* genomes [43]. These putative self-targeting CRISPR spacers in *M. bovis* genomes may indicate the presence of *Acrs* in *M. bovis*. Further study is needed to identify and characterize any potential *M. bovis Acrs*.

Within the environment of a mammalian host, iron is limiting. Competition for iron shapes interactions between pathogens and hosts, between pathogens and other pathogens, and between pathogens and commensals [44]. Iron repressible OMPs (IrpA) and iron acquisition genes have been previously described in *M. bovis* [45, 46]. Many of the predicted outer membrane proteins identified in this study are involved in iron acquisition and transport. TonB dependent receptors were identified in some genotype 2 *M. bovis* prophages versus none in genotype 1 prophages, however, we also identified a TonB dependent receptor that is common to all genomes of *M. bovis* as well as all genomes of genotype 1 *M. bovoculi* but is absent from all genomes of genotype 2 *M. bovoculi*. Conversely, several TonB dependent receptors and other iron transporting proteins have been identified in all genomes of genotype 2 *M. bovoculi* but are absent from all genomes of *M. bovis* as well as genotype 1 *M. bovoculi*. Genes involved in iron piracy have long been known to be virulence factors in numerous pathogenic bacteria [44, 47, 48]. While samples of both genotypes of *M. bovis* and genotype 1 *M. bovoculi* are commonly collected from animals showing signs of disease, samples of genotype 2 *M. bovoculi* have only been collected from healthy animals to our knowledge [10, 23]. It is possible that the differences in genes that code for iron piracy proteins is a determinant in the differences in pathogenicity between these groups. Further study is needed to elucidate the effects of iron piracy on niche partitioning within the bovine eye. The gene coding for the TonB dependent receptor common to both genotypes of *M. bovis* and genotype 1 *M. bovoculi* is highly conserved. When comparing the translated sequence of all samples, the full-length protein has a minimum similarity of 93.8% and the predicted EL regions have a minimum similarity of 97.5%. Due to its presence only in the genomes of the genotypes associated with disease as well as the high sequence conservation, this TonB dependent receptor is particularly interesting for the development of future intervention strategies.

## Conclusions

Two major genotypes of *M. bovis* were identified in this study through whole genome sequencing. The genotypes differ by their associations with plasmids (including plasmid type 1 which can carry one or two copies of genes encoding haemagglutinin-like proteins), hemolysin and pilin protein variants, prophage content, and by their core genomes. Based on potential virulence factor frequencies and protein differences, the two *M. bovis* genotypes may vary in their pathogenesis or associations with IBK, and that should be experimentally tested. Recombination may play a significant role in *M. bovis* evolution and immune evasion as evidenced by apparent recombination in both hemolysin and pilin genes. Immunological reagents can be developed to target both *M. bovis* genotypes, or assortments of *M. bovis* and *M. bovoculi* genotypes.

## Methods

### Assembly of a diverse collection of *M. bovis* strains

Thirty-six strains of *M. bovis* were selected for sequencing and whole genome assembly that represented seventeen U.S. States and one Canadian province (Supplementary File S1). Each of the strains were isolated from cattle between 1978 and 2020. The identity of the strains was determined by combinations of colony morphology following growth on tryptic soy agar with 5% sheep’s blood, positive oxidase activity, and a negative Gram stain with coccobacillus morphology. Additionally, strains isolated prior to 2017 were subjected to a PCR-RFLP designed to differentiate *Moraxella ovis*, *M. bovoculi*, and *M. bovis* [13, 49]. For the PCR-RFLP, a small portion of the 16S gene, the entire 16S–23S intergenic spacer, and a small portion of the 23S gene was amplified [13, 49]. The *M. bovis* amplicon was approximately 650 bp and did not cleave from digestion with *Afa*I [13, 49]. Additional tests to those mentioned above were done on some of the strains where necessary [13]. The identity of all 36 strains was also confirmed through direct smear analysis using matrix assisted laser desorption ionization time-of-flight mass spectrometry (MALDI-TOF MS) as previously described [50].

### Culture conditions, DNA purification, library construction and sequencing

All 36 strains were revived from frozen stock vials by two passages on chocolate agar plates with 1% bovine hemoglobin and growth/nutrient supplements (Hardy Diagnostics, Santa Maria, CA, USA). The plates were incubated at 37 °C with 5% CO_2_. Due to strain differences in growth rates, single colonies were transferred to liquid brain heart infusion (BHI) broth (pH 7.3) in volumes ranging from 2 mL to 50 mL and cultured at 37 °C with 5% CO_2_ and shaking for several hours or for several days until mid-log growth was observed. Two mL starter cultures were used to seed 20 mL–50 mL of BHI, which were then cultured at 37 °C with 5% CO_2_ until mid-log growth was observed. Either a GENESYS 20 (Waltham, MA, USA) or a DeNovix DS-11FX+ spectrophotometer (Wilmington, DE, USA) was used to assess *M. bovis* growth in liquid BHI.

Strain DNAs were extracted and purified on Qiagen 100/G gravity-flow anion-exchange columns (Qiagen, Valencia, CA, USA) as previously described [51]. The DNAs were quantified using either a Promega Quantus Fluorometer and QuantiFluor Dyes per the manufacturer’s instructions (Promega, Madison, WI, USA), or with a DeNovix DS-11FX+ spectrophotometer (Wilmington, DE, USA). For PacBio sequencing, single molecule real-time DNA libraries (SMRT Bell 1.0, 10–20 kBP) were constructed for each strain and sequenced on either an RS II or Sequel instrument according to the manufacturer’s instructions (Menlo Park, CA, USA). For Illumina sequencing, TruSeq DNA-PCR free libraries were constructed for a majority of the strains according to the manufacturer’s instructions (San Diego, CA, USA). For several strains, RipTide libraries were constructed according to the manufacturer’s instructions (iGenomX, San Diego, CA, USA). The libraries were sequenced on either a MiSeq or NextSeq 500 instrument.

### Whole genome sequencing and assembly

For each strain, the PacBio reads were assembled de novo into initial chromosome and plasmid contigs using PacBio’s Hierarchical Genome Assembly Process (HGAP 4) assembly software within the SMRT Link software package (versions 6–9). PacBio sequence reads were then mapped to the initially assembled contigs using Geneious Prime software (versions 2019.0–2021.2), (Auckland, NZ) and scanned for overlapping regions on each end to check for correct circularization of the contigs by the assembly software. For strain SAM109242, two chromosomal contigs were manually merged together using mapped PacBio reads anchored to unique sequence within each contig (GenBank accession number CP087776). Additionally, tandem repeat motif regions in all of the strain chromosomes were manually checked to ensure they were represented by actual reads that spanned the regions. The Illumina reads were also mapped to the assembled chromosomes and plasmids to check for and correct any homopolymer errors that may have occurred from the PacBio sequencing. Searches for origins of replication within the chromosomes were performed using ORI-Finder 1 [52, 53], and the chromosomes were orientated to start at the strongest supported ORI.

To identify plasmids, particularly small ones that could have been missed on the PacBio platform, Illumina reads that did not map to PacBio generated chromosomes or plasmids were assembled de novo in Geneious. Resulting contigs from those assemblies were scanned for overlapping sequences on each end to indicate the contigs could be circularized and subjected to BLAST searches for identification. The plasmids were aligned with Mauve in Geneious to identify regions of homology, and thus different plasmid types. If a representative of a plasmid type was identified in the NCBI database, all plasmids of that type were orientated to the start of the reference plasmid. Plasmids without representatives in NCBI were orientated to their assembly configurations.

### Phylogenetic tree builds

A Neighbor-Joining tree of the 36 *M. bovis* genomes sequenced in this study, along with one available completely assembled genome of *M. bovis* strain Epp63 in GenBank and seven of *M. bovoculi* was constructed in EDGAR (version 3.0, https://edgar3.computational.bio.uni-giessen.de/cgi-bin/edgar.cgi), [54]. EDGAR first computed core genes for the set of isolates and then aligned them with MUSCLE [55, 56]. The alignments were concatenated, and the tree was generated by EDGAR with a F84 model of substitution using PHYLIP [57]. For the tree build, EDGAR computed a core of 1220 genes and an alignment of 1,278,792 bases per genome. The tree was viewed with Dendroscope 3 (version 3.5.10) [58].

To confirm phylogenetic relationships, whole genome trees of the 37 *M bovis* strains were additionally constructed with Parsnp (Harvest version 1.1.2) [59]. As previously described elsewhere [51], Parsnp identifies locally colinear blocks of multi-maximal unique matches which are used to anchor multiple alignments [59]. MUSCLE was used for generating the alignments [55, 56]. An approximately maximum likelihood phylogenetic tree was generated with FastTree 2 with a Jukes Cantor model of substitution from an alignment of polymorphisms that passed filtering criteria for quality, possible recombination, and other parameters [60, 61]. For the tree build, the Parsnp multi-MUM search and libMUSCLE aligner was run, as was a reconstruction of core genome phylogeny, and the creation of a Gingr input file. *M. bovis* Epp63 was used as a reference with 74% of the genome incorporated into the tree build. The tree was viewed in either Gingr (version 1.1.1) [59] or Dendroscope 3 (version 3.5.10) [58]. A variant call file from the tree build was used to identify SNPs, determine their location on Epp63 reference sequence, and score SNP alleles for each *M. bovis* strain.

### Average nucleotide identities, tandem repeat and prophage identifications

Average Nucleotide Identities (ANIs) were calculated for the 37 *M. bovis* strains and seven *M. bovoculi* strains with fully assembled genomes (Supplementary File S1) in JSpeciesWS using Blast+ calculations (web server accessed between 12/21 and 4/22), [62]. The incompletely assembled genome sequence of *M. bovis* strain CCUG 2133 was used as a reference for the ANI calculations as it is a type strain representative of the species (https://www.ncbi.nlm.nih.gov/biosample/SAMN06308688). Tandem repeats were identified within bacterial genomes using Tandem repeats finder (version 4.10.0) [63], and prophages were identified with the web version of Phaster [64, 65].

### Identification of pan and core genomes of *M. bovis* and *M. bovoculi*, and predicted outer membrane proteins

Whole genomes of *M. bovis* (*n* = 1) and *M. bovoculi* (*n* = 7) that were isolated from cattle were downloaded from GenBank on 4/28/21. To ensure consistent annotation, all downloaded genomes and all newly sequenced genomes were annotated with DFAST (v1.2.6) [66]. To identify homologous coding sequences across species, a pan-genome analysis was done with EDGAR (v3.0) [54] using *M. bovis* genotype 2 genome CP087830 as a reference. The pan-genome analysis output was sorted to extract the core genome of each individual species and the core genome shared between species; a 95% cutoff was applied to be considered core. Amino acid sequences of the core genomes were analyzed by BOMP (web server accessed between 05/21 and 10/21) [67] to identify beta-barrel motifs and predict outer membrane proteins. Coding sequences that were predicted to be localized to the outer membrane were then analyzed by Boctopus2 (web server accessed between 05/21 and 10/21) [68] to confirm the outer membrane prediction as well as to identify the specific amino acids likely to be found on the outer membrane. Python scripts were written and used to extract amino acid sequences from sorted EDGAR output (https://github.com/EmilyWynn/EDGAR-protein-grabber). To measure the similarity of the predicted OMPs across species, alignments were made using a BLOSUM62 scoring matrix for both the full-length proteins as well as their predicted EL domains.

### Identification of pilin and hemolysin genes and proteins

To identify and categorize protein variants encoded by *pilA*, fimbrial proteins were identified from DFAST annotation. Regions downstream of *pilA* were extracted from fasta files and translated with Expasy Translate (https://web.expasy.org/translate/) to identify inverted fimbrial protein sequence. Alignments of forward orientation *pilA* and downstream, inverted orientations of *pilA* were generated in Geneious and tested for recombination using a Phi test in SplitsTree (version 4.16.1) [61, 69]. Translated *pilA* amino acid sequences produced from this study were aligned with *pilA* amino acid sequences of serogroup reference strains [25] in Geneious. Sequences were considered the same protein variant if they had greater than 95% pairwise sequence similarity using BLOSUM62 scoring [70]. To identify protein variants of hemolysin, the gene for each strain was located using NCBI annotation and both gene and corresponding amino acid sequences were extracted and aligned in MacVector (version 18.2.5). Unique hemolysin gene sequences (*n* = 3) were tested for recombination using a Phi test in SplitsTree (version 4.16.1) [61, 69].

### Identification of CRISPR/Cas systems and antimicrobial resistance genes

To identify CRISPRs and Cas genes, all genomes were analyzed by CRISPRCasFinder (web server accessed 11/21) [71]. To identify potential CRISPR self-targeting, CRISPR spacers were used as a query for a BLAST search of the *M. bovis* genome. BLAST hits within the CRISPR region itself were excluded and BLAST hits from other regions of the genome were noted. To identify antimicrobial resistance genes, all genomes were analyzed by ResFinder (v4.0) with a 90% threshold for ID and a 60% minimum length [72–74, 75].

## Supplementary Information

**Additional file 1.** 

**Additional file 2.** 

**Additional file 3.**

**Additional file 4.**

**Additional file 5.** 

**Additional file 6.**

**Additional file 7.**

**Additional file 8.**

**Additional file 9.**

**Additional file 10.**

**Additional file 11.**

**Additional file 12.**

**Additional file 13.**

**Additional file 14.**

## Data Availability

All assembled chromosomes and plasmids, as well as Illumina fastq files and PacBio BAM files have been made publicly available in NCBI. See Supplementary File S1 for a complete listing of all accession file numbers deposited in NCBI from this study and for the accession numbers of previously published genomes that were also included in this study.

## Abbreviations

AMR: Antimicrobial resistance
ANI: Average nucleotide identity
ARS: Agricultural Research Service
BHI: Brain heart infusion
BLAST: Basic Local Alignment Search Tool
C: Celsius
CO_2_: Carbon dioxide
CRISPR: Clustered regularly interspaced short palindromic repeats
DNA: Deoxyribonucleic acid
EL: extracellular loop
HGAP: Hierarchical Genome Assembly Process
IBK: Infectious bovine keratoconjunctivitis
kbp: 1000 base pairs
MALDI-TOF MS: matrix assisted laser desorption ionization time-of-flight mass spectrometry
*M. bovis*: *Moraxella bovis*
*M. bovoculi*: *Moraxella bovoculi*
mL: milliliters
NCBI: National Center for Biotechnology Information
NVDC: Nebraska Veterinary Diagnostic Center
OMP: Outer membrane protein
ORI: Origin of replication
PADase: phenylalanine deaminase
pH: Potential hydrogen
PCR: Polymerase chain reaction
RTX: Repeats in toxin
SNP: Single nucleotide polymorphism
UNL: University of Nebraska-Lincoln
USDA: United States Department of Agriculture

## References

[1] Dennis EJ, Kneipp M (2021). A review of global prevalence and economic impacts of infectious bovine Keratoconjunctivitis. Vet Clin North Am Food Anim Pract..

[2] Seid A (2019). Review on infectious bovine Keratoconjunctivitis and its economic impacts in cattle. Journal of Dairy & Veterinary Sciences.

[3] Comin HB, Sollero BP, Gapar EB, Domingues R, Cardoso FF (2021). Genome-wide association study of resistance/susceptibility to infectious bovine keratoconjunctivitis in Brazilian Hereford cattle. Anim Genet.

[4] O'Connor AM (2021). Component causes of infectious bovine Keratoconjunctivitis: the role of genetic factors in the epidemiology of infectious bovine Keratoconjunctivitis. Vet Clin North Am Food Anim Pract..

[5] Defining KM, Keratoconjunctivitis DIB (2021). Vet Clin North Am Food Anim Pract..

[6] Killinger AH, Valentine D, Mansfield ME, Ricketts GE, Cmarik GF, Neumann AH (1977). Economic-impact of infectious-bovine-Keratoconjunctivitis in beef-calves. Vet Med Sm Anim Clin.

[7] Dewell RD, Millman ST, Gould SA, Tofflemire KL, Whitley RD, Parsons RL (2014). Evaluating approaches to measuring ocular pain in bovine calves with corneal scarification and infectious bovine keratoconjunctivitis-associated corneal ulcerations. J Anim Sci.

[8] Cameron A, McAllister TA (2016). Antimicrobial usage and resistance in beef production. J Anim Sci Biotechnol.

[9] O'Connor AM, Kneipp M (2021). Evidence base for treatment of infectious bovine Keratoconjunctivitis. Vet Clin North Am Food Anim Pract..

[10] Dickey AM, Schuller G, Loy JD, Clawson ML (2018). Whole genome sequencing of *Moraxella bovoculi* reveals high genetic diversity and evidence for interspecies recombination at multiple loci. PLoS One.

[11] Loy JD, Hille M, Maier G, Clawson ML (2021). Component causes of infectious bovine Keratoconjunctivitis - the role of *Moraxella* species in the epidemiology of infectious bovine Keratoconjunctivitis. Vet Clin North Am Food Anim Pract.

[12] Loy JD, Clothier KA, Maier G (2021). Component causes of infectious bovine Keratoconjunctivitis-non-Moraxella organisms in the epidemiology of infectious bovine Keratoconjunctivitis. Vet Clin North Am Food Anim Pract..

[13] Loy JD, Brodersen BW (2014). Moraxella spp. isolated from field outbreaks of infectious bovine keratoconjunctivitis: a retrospective study of case submissions from 2010 to 2013. J Vet Diagn Investig.

[14] Rogers DG, Cheville NF, Pugh GW (1987). Conjunctival lesions caused by *Moraxella bovis* in gnotobiotic calves. Vet Pathol.

[15] Rogers DG, Cheville NF, Pugh GW (1987). Pathogenesis of corneal lesions caused by *Moraxella bovis* in gnotobiotic calves. Vet Pathol.

[16] Beard MK, Moore LJ (1994). Reproduction of bovine keratoconjunctivitis with a purified haemolytic and cytotoxic fraction of *Moraxella bovis*. Vet Microbiol.

[17] Marrs CF, Ruehl WW, Schoolnik GK, Falkow S (1988). Pilin-gene phase variation of *Moraxella bovis* is caused by an inversion of the pilin genes. J Bacteriol.

[18] Moore LJ, Lepper AW (1991). A unified serotyping scheme for *Moraxella bovis*. Vet Microbiol.

[19] Kakuda T, Sarataphan N, Tanaka T, Takai S (2006). Filamentous-haemagglutinin-like protein genes encoded on a plasmid of *Moraxella bovis*. Vet Microbiol.

[20] Pugh GW, McDonald TJ (1986). Identification of bovine carriers of *Moraxella bovis* by comparative cultural examinations of ocular and nasal secretions. Am J Vet Res.

[21] Bartenslager AC, Althuge ND, Loy JD, Hille MM, Spangler ML, Fernando SC (2021). Longitudinal assessment of the bovine ocular bacterial community dynamics in calves. Animal Microbiome.

[22] Gould S, Dewell R, Tofflemire K, Whitley RD, Millman ST, Opriessnig T (2013). Randomized blinded challenge study to assess association between *Moraxella bovoculi* and infectious bovine Keratoconjunctivitis in dairy calves. Vet Microbiol.

[23] Dickey AM, Loy JD, Bono JL, Smith TP, Apley MD, Lubbers BV (2016). Large genomic differences between *Moraxella bovoculi* isolates acquired from the eyes of cattle with infectious bovine keratoconjunctivitis versus the deep nasopharynx of asymptomatic cattle. Vet Res.

[24] Loy JD, Dickey AM, Clawson ML. Complete genome sequence of *Moraxella bovis* strain Epp-63 (300), an etiologic agent of infectious bovine Keratoconjunctivitis. Microbiol Resour Announc. 2018;7(8). 10.1128/MRA.01004-18. 10.1128/MRA.01004-18 PMC6256509 30533917

[25] Atwell JL, Tennent JM, Lepper AW, Elleman TC (1994). Characterization of pilin genes from seven serologically defined prototype strains of *Moraxella bovis*. J Bacteriol.

[26] Torresen OK, Star B, Mier P, Andrade-Navarro MA, Bateman A, Jarnot P (2019). Tandem repeats lead to sequence assembly errors and impose multi-level challenges for genome and protein databases. Nucleic Acids Res.

[27] Schuster CF, Bertram R. Toxin-antitoxin systems of *Staphylococcus aureus*. *Toxins* (Basel). 2016;8(5). 10.3390/toxins8050140. 10.3390/toxins8050140 PMC4885055 27164142

[28] Novick RP (1987). Plasmid incompatibility. Microbiol Rev.

[29] Klemm P, Schembri MA (2000). Bacterial adhesins: function and structure. Int J Med Microbiol.

[30] Orndorff PE, Cotter Peggy A, Yuk Ming H, Mattoo S, Akerley Brian J, Boschwitz J (1998). Filamentous hemagglutinin of *Bordetella bronchiseptica* is required for efficient establishment of tracheal colonization. Infect Immun.

[31] Angelos JA, Ball LM, Hess JF (2007). Identification and characterization of complete RTX operons in *Moraxella bovoculi* and *Moraxella ovis*. Vet Microbiol.

[32] Angelos JA, Hess JF, George LW (2003). An RTX operon in hemolytic *Moraxella bovis* is absent from nonhemolytic strains. Vet Microbiol.

[33] Pugh GW, Hughes DE (1970). Comparison of the virulence of various strains of *Moraxella bovis*. Can J Comp Med.

[34] Pugh GW, Hughes DE (1968). Experimental bovine infectious keratoconjunctivitis caused by sunlamp irradiation and *Moraxella bovis* infection: correlation of hamolytic ability and pathogenicity. Am J Vet Res.

[35] Angelos JA, Hess JF, George LW (2001). Cloning and characterization of a *Moraxella bovis* cytotoxin gene. Am J Vet Res.

[36] Jayappa HG, Lehr C (1986). Pathogenicity and immunogenicity of piliated and nonpiliated phases of *Moraxella bovis* in calves. Am J Vet Res.

[37] Lepper AW, Moore LJ, Atwell JL, Tennent JM (1992). The protective efficacy of pili from different strains of *Moraxella bovis* within the same serogroup against infectious bovine keratoconjunctivitis. Vet Microbiol.

[38] Lepper AW, Elleman TC, Hoyne PA, Lehrbach PR, Atwell JL, Schwartzkoff CL (1993). A *Moraxella bovis* pili vaccine produced by recombinant DNA technology for the prevention of infectious bovine keratoconjunctivitis. Vet Microbiol.

[39] Angelos JA, Gohary KG, Ball LM, Hess JF (2012). Randomized controlled field trial to assess efficacy of a *Moraxella bovis* pilin-cytotoxin-*Moraxella bovoculi* cytotoxin subunit vaccine to prevent naturally occurring infectious bovine keratoconjunctivitis. Am J Vet Res.

[40] Lepper AWD, Atwell JL, Lehrbach PR, Schwartzkoff CL, Egerton JR, Tennent JM (1995). The protective efficacy of cloned *Moraxella-Bovis* pili in monovalent and multivalent vaccine formulations against experimentally-induced infectious bovine Keratoconjunctivitis (Ibk). Vet Microbiol.

[41] Raadsma HW, O'Meara TJ, Egerton JR, Lehrbach PR, Schwartzkoff CL (1994). Protective antibody titres and antigenic competition in multivalent *Dichelobacter nodosus* fimbrial vaccines using characterised rDNA antigens. Vet Immunol Immunopathol.

[42] Shmakov SA, Sitnik V, Makarova KS, Wolf YI, Severinov KV, Koonin EV. The CRISPR spacer space is dominated by sequences from species-specific Mobilomes. mBio. 2017;8(5). 10.1128/mBio.01397-17. 10.1128/mBio.01397-17 PMC5605939 28928211

[43] Marino ND, Zhang JY, Borges AL, Sousa AA, Leon LM, Rauch BJ (2018). Discovery of widespread type I and type V CRISPR-Cas inhibitors. Science..

[44] Barber MF, Elde NC (2015). Buried treasure: evolutionary perspectives on microbial Iron piracy. Trends Genet.

[45] Kakuda T, Oishi D, Tsubaki S, Takai S (2003). Molecular cloning and characterization of a 79-kDa iron-repressible outer-membrane protein of *Moraxella bovis*. FEMS Microbiol Lett.

[46] Kakuda T, Oishi D, Tsubaki S, Takai S (2003). Cloning and characterization of the fur gene from *Moraxella bovis*. Microbiol Immunol.

[47] Wooldridge KG, Williams PH (1993). Iron uptake mechanisms of pathogenic bacteria. FEMS Microbiol Rev.

[48] Ratledge C (2007). Iron metabolism and infection. Food Nutr Bull.

[49] Angelos JA, Ball LM (2007). Differentiation of *Moraxella bovoculi* sp nov from other coccoid moraxellae by the use of polymerase chain reaction and restriction endonuclease analysis of amplified DNA. J Vet Diagn Investig.

[50] Robbins K, Dickey AM, Clawson ML, Loy JD (2018). Matrix-assisted laser desorption/ionization time-of-flight mass spectrometry identification of *Moraxella bovoculi* and *Moraxella bovis* isolates from cattle. J Vet Diagn Investig.

[51] Clawson ML, Schuller G, Dickey AM, Bono JL, Murray RW, Sweeney MT (2020). Differences between predicted outer membrane proteins of genotype 1 and 2 *Mannheimia haemolytica*. BMC Microbiol.

[52] Luo H, Quan CL, Peng C, Gao F (2019). Recent development of Ori-finder system and DoriC database for microbial replication origins. Brief Bioinform.

[53] Gao F, Zhang CT (2008). Ori-finder: a web-based system for finding oriCs in unannotated bacterial genomes. BMC Bioinformatics..

[54] Blom J, Kreis J, Spänig S, Juhre T, Bertelli C, Ernst C (2016). EDGAR 2.0: an enhanced software platform for comparative gene content analyses. Nucleic Acids Res.

[55] Edgar RC (2004). MUSCLE: a multiple sequence alignment method with reduced time and space complexity. BMC Bioinformatics.

[56] Edgar RC (2004). MUSCLE: multiple sequence alignment with high accuracy and high throughput. Nucleic Acids Res.

[57] Felsenstein J (1989). PHYLIP — phylogeny inference package (version 3.2). Cladistics..

[58] Huson DH, Scornavacca C. Dendroscope 3: an interactive tool for rooted phylogenetic trees and networks. Syst Biol. 2012. 10.1093/sysbio/sys062 22780991

[59] Treangen TJ, Ondov BD, Koren S, Phillippy AM (2014). The harvest suite for rapid core-genome alignment and visualization of thousands of intraspecific microbial genomes. Genome Biol.

[60] Price MN, Dehal PS, Arkin AP (2010). FastTree 2--approximately maximum-likelihood trees for large alignments. PLoS One.

[61] Bruen TC, Philippe H, Bryant D (2006). A simple and robust statistical test for detecting the presence of recombination. Genetics..

[62] Richter M, Rossello-Mora R, Oliver Glockner F, Peplies J (2016). JSpeciesWS: a web server for prokaryotic species circumscription based on pairwise genome comparison. Bioinformatics..

[63] Benson G (1999). Tandem repeats finder: a program to analyze DNA sequences. Nucleic Acids Res.

[64] Zhou Y, Liang Y, Lynch KH, Dennis JJ, Wishart DS (2011). PHAST: a fast phage search tool. Nucleic Acids Res.

[65] Arndt D, Grant JR, Marcu A, Sajed T, Pon A, Liang Y (2016). PHASTER: a better, faster version of the PHAST phage search tool. Nucleic Acids Res.

[66] Tanizawa Y, Fujisawa T, Nakamura Y (2018). DFAST: a flexible prokaryotic genome annotation pipeline for faster genome publication. Bioinformatics..

[67] Berven FS, Flikka K, Jensen HB, Eidhammer I (2004). BOMP: a program to predict integral beta-barrel outer membrane proteins encoded within genomes of gram-negative bacteria. Nucleic Acids Res.

[68] Hayat S, Peters C, Shu N, Tsirigos KD, Elofsson A (2016). Inclusion of dyad-repeat pattern improves topology prediction of transmembrane β-barrel proteins. Bioinformatics..

[69] Huson DH, Bryant D (2006). Application of phylogenetic networks in evolutionary studies. Mol Biol Evol.

[70] Angelos JA, Clothier KA, Agulto RL, Mandzyuk B, Tryland M. Relatedness of type IV pilin PilA amongst geographically diverse *Moraxella bovoculi* isolated from cattle with infectious bovine keratoconjunctivitis. J Med Microbiol. 2021;70(2). 10.1099/jmm.0.001293. 10.1099/jmm.0.001293 PMC8131017 33404383

[71] Couvin D, Bernheim A, Toffano-Nioche C, Touchon M, Michalik J, Néron B (2018). CRISPRCasFinder, an update of CRISRFinder, includes a portable version, enhanced performance and integrates search for Cas proteins. Nucleic Acids Res.

[72] Bortolaia V, Kaas RS, Ruppe E, Roberts MC, Schwarz S, Cattoir V (2020). ResFinder 4.0 for predictions of phenotypes from genotypes. J Antimicrob Chemoth.

[73] Camacho C, Coulouris G, Avagyan V, Ma N, Papadopoulos J, Bealer K, et al. BLAST plus : architecture and applications. BMC Bioinformatics. 2009;10. 10.1186/1471-2105-10-421. 10.1186/1471-2105-10-421 PMC2803857 20003500

[74] Zankari E, Allesoe R, Joensen KG, Cavaco LM, Lund O, Aarestrup FM (2017). PointFinder: a novel web tool for WGS-based detection of antimicrobial resistance associated with chromosomal point mutations in bacterial pathogens. J Antimicrob Chemoth..

[75] Camacho C, Coulouris G, Avagyan V, Ma N, Papadopoulos J, Bealer K (2009). BLAST+: architecture and applications. BMC Bioinformatics..

