# Supplementary material for: Whole genome sequencing of Moraxella bovis strains from North America reveals two genotypes with different genetic determinants
Source: BMC Microbiol. 2022 Oct 21;22:258. doi: 10.1186/s12866-022-02670-3 (PMC9585708; doi:10.1186/s12866-022-02670-3)
Supplement: Supplementary file 1 — Additional file 1. [file 12866_2022_2670_MOESM1_ESM.ppt]

## Slide 1
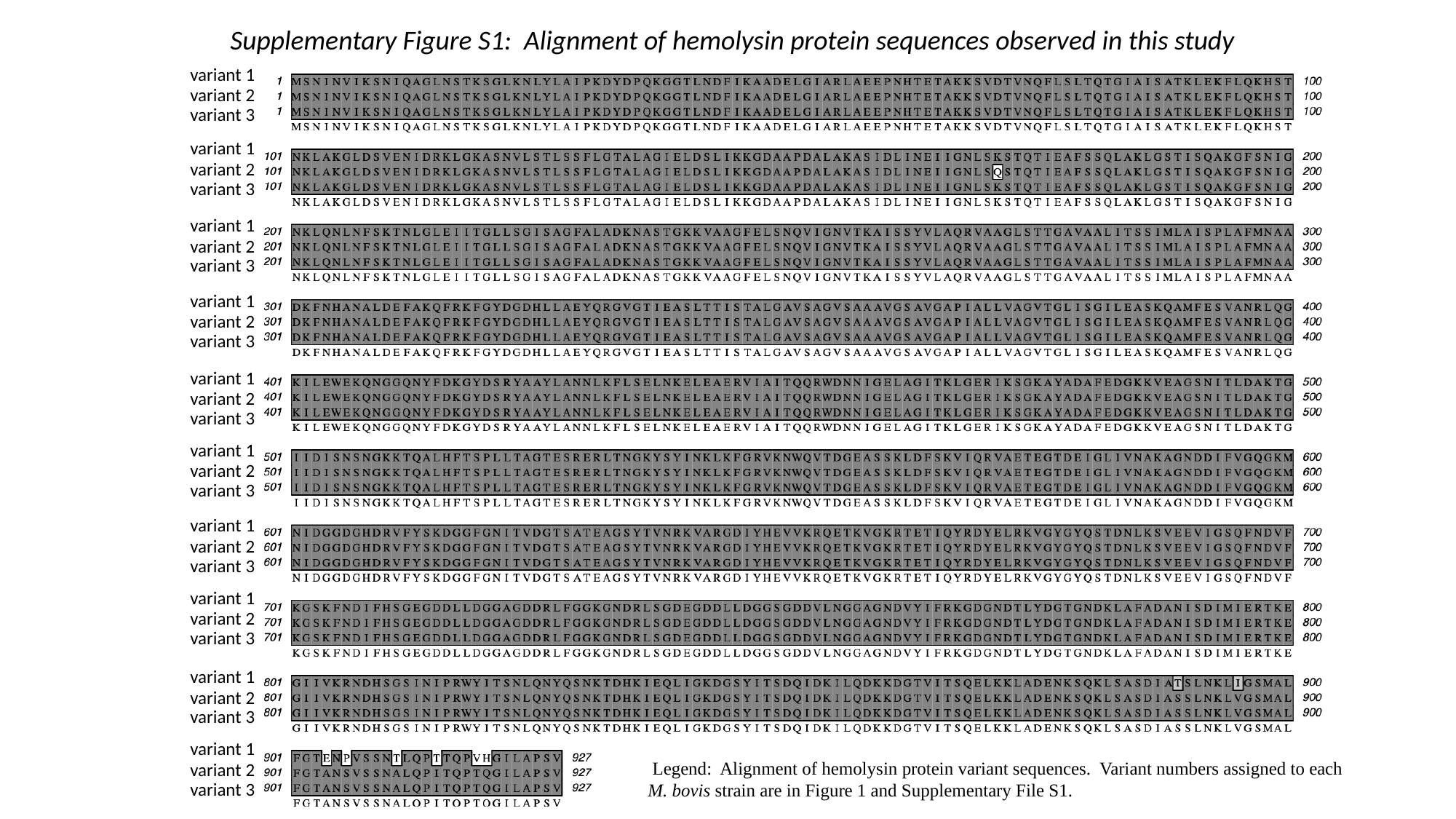

Supplementary Figure S1: Alignment of hemolysin protein sequences observed in this study
variant 1
variant 2
variant 3
variant 1
variant 2
variant 3
variant 1
variant 2
variant 3
variant 1
variant 2
variant 3
variant 1
variant 2
variant 3
variant 1
variant 2
variant 3
variant 1
variant 2
variant 3
variant 1
variant 2
variant 3
variant 1
variant 2
variant 3
variant 1
 Legend: Alignment of hemolysin protein variant sequences. Variant numbers assigned to each M. bovis strain are in Figure 1 and Supplementary File S1.
variant 2
variant 3
