# Supplementary material for: Whole genome sequencing of Moraxella bovis strains from North America reveals two genotypes with different genetic determinants
Source: BMC Microbiol. 2022 Oct 21;22:258. doi: 10.1186/s12866-022-02670-3 (PMC9585708; doi:10.1186/s12866-022-02670-3)
Supplement: Supplementary file 2 — Additional file 2. [file 12866_2022_2670_MOESM2_ESM.ppt]

## Slide 1
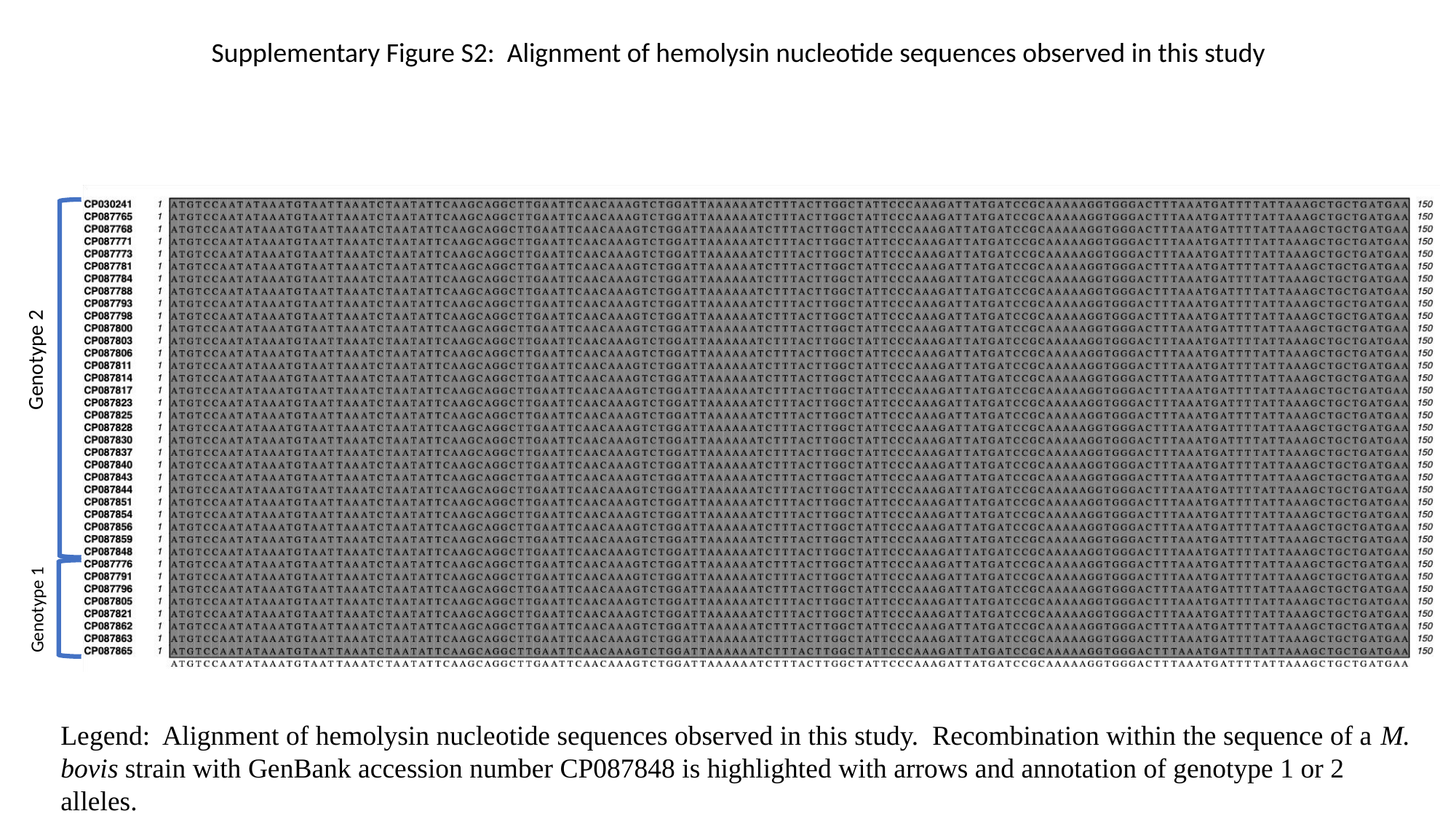

Supplementary Figure S2: Alignment of hemolysin nucleotide sequences observed in this study
Genotype 2
Genotype 1
Legend: Alignment of hemolysin nucleotide sequences observed in this study. Recombination within the sequence of a M. bovis strain with GenBank accession number CP087848 is highlighted with arrows and annotation of genotype 1 or 2 alleles.

## Slide 2
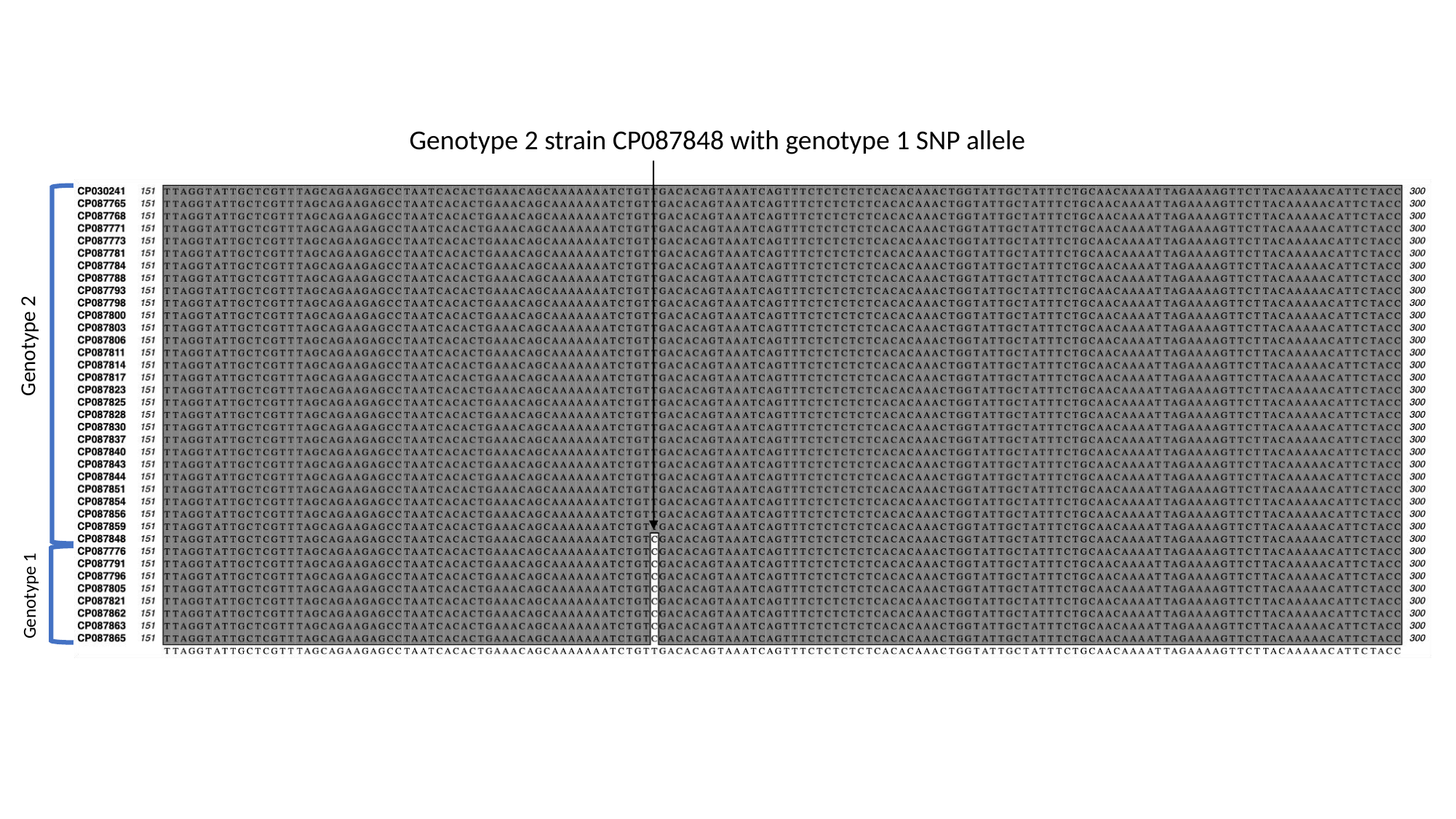

Genotype 2 strain CP087848 with genotype 1 SNP allele
Genotype 2
Genotype 1

## Slide 3
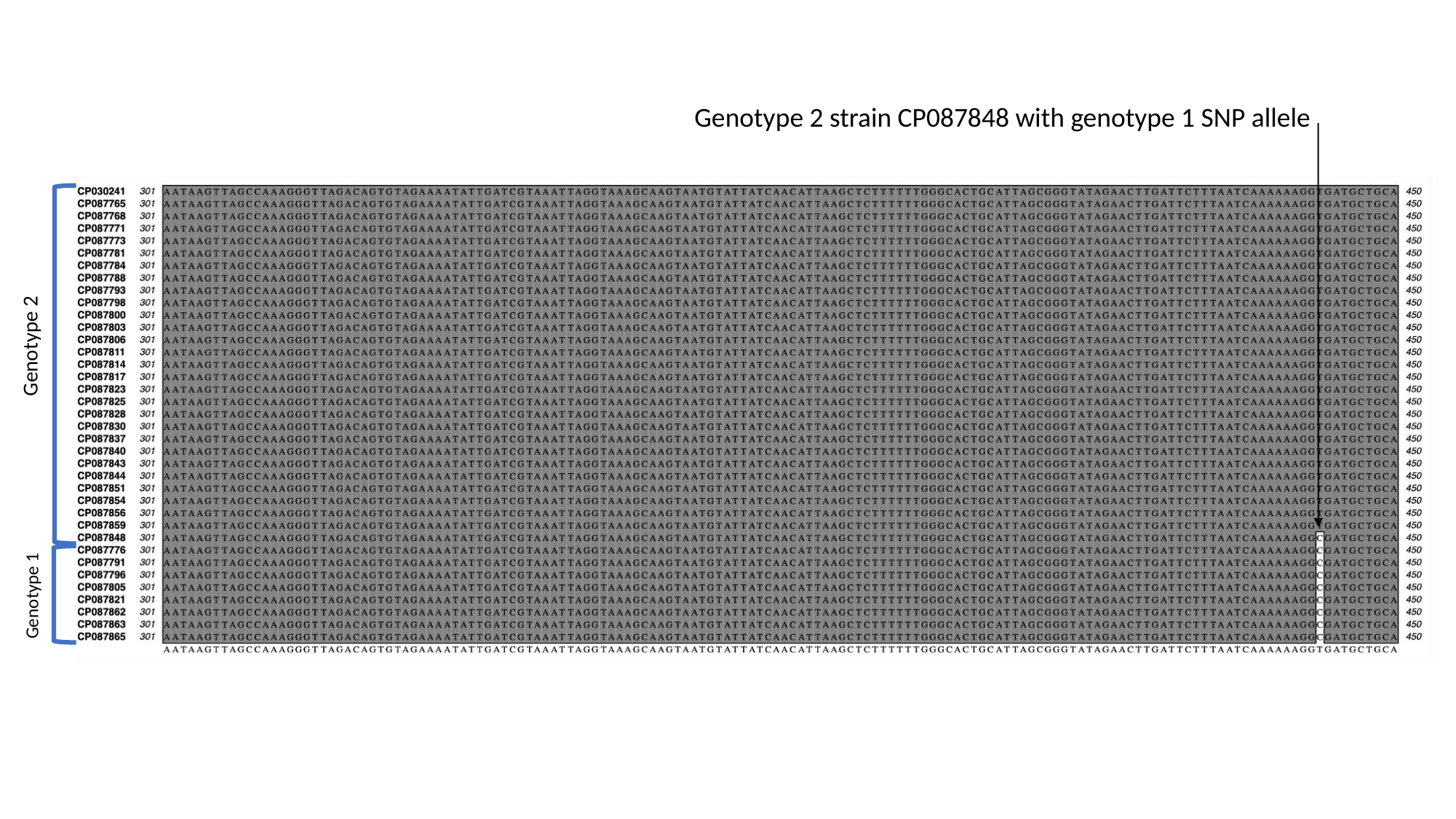

Genotype 2 strain CP087848 with genotype 1 SNP allele
Genotype 2
Genotype 1

## Slide 4
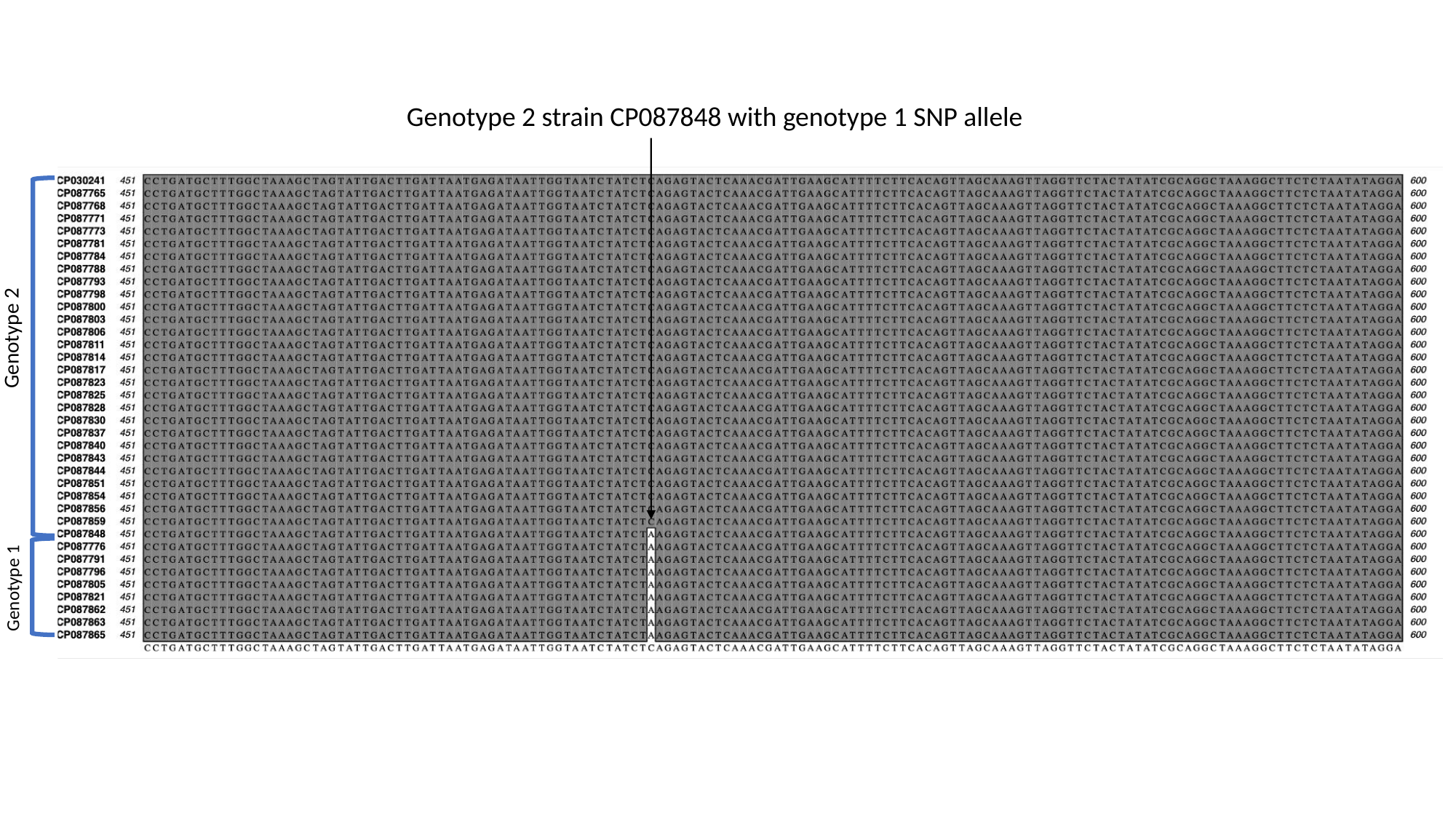

Genotype 2 strain CP087848 with genotype 1 SNP allele
Genotype 2
Genotype 1

## Slide 5
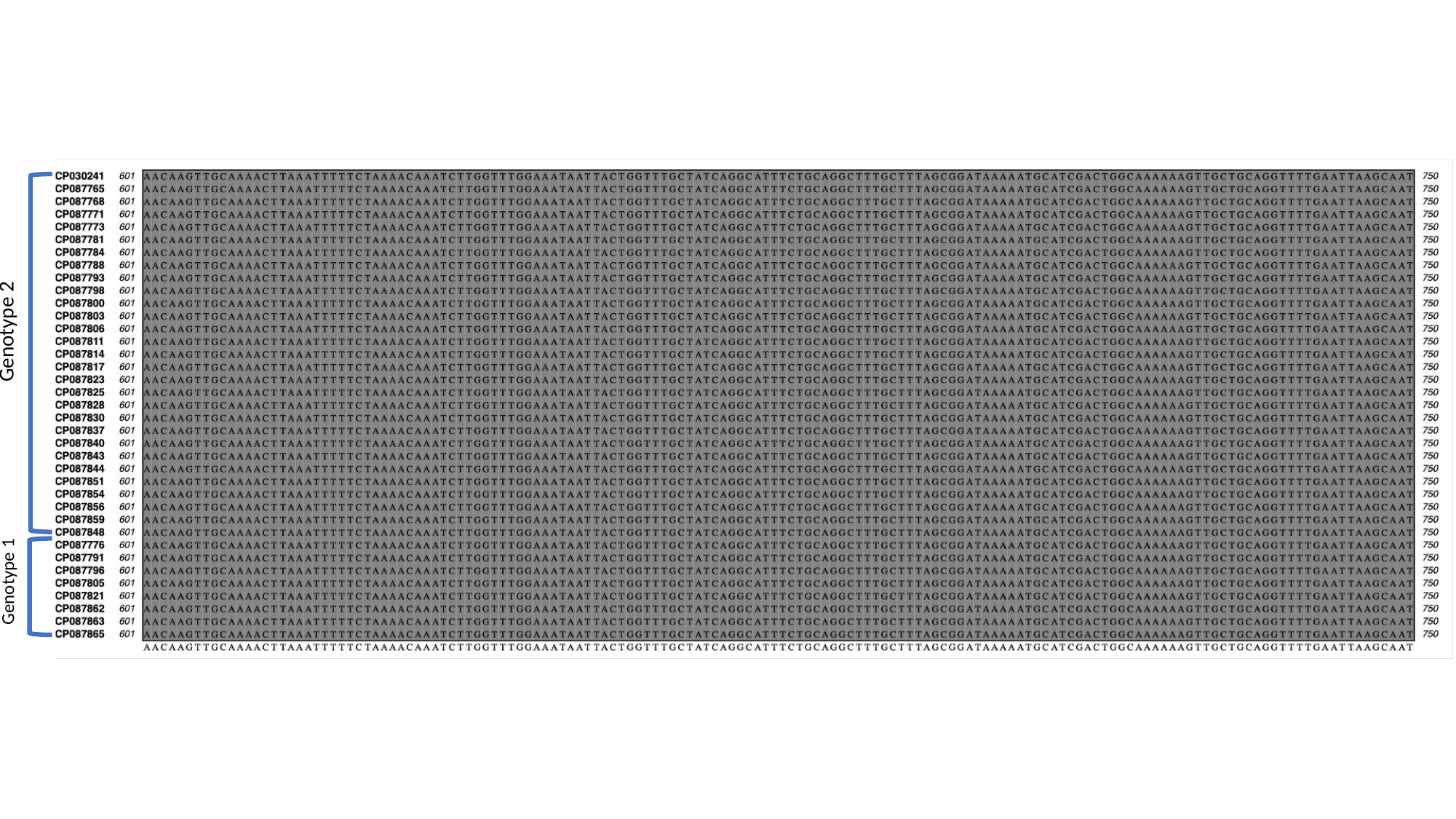

Genotype 2
Genotype 1

## Slide 6
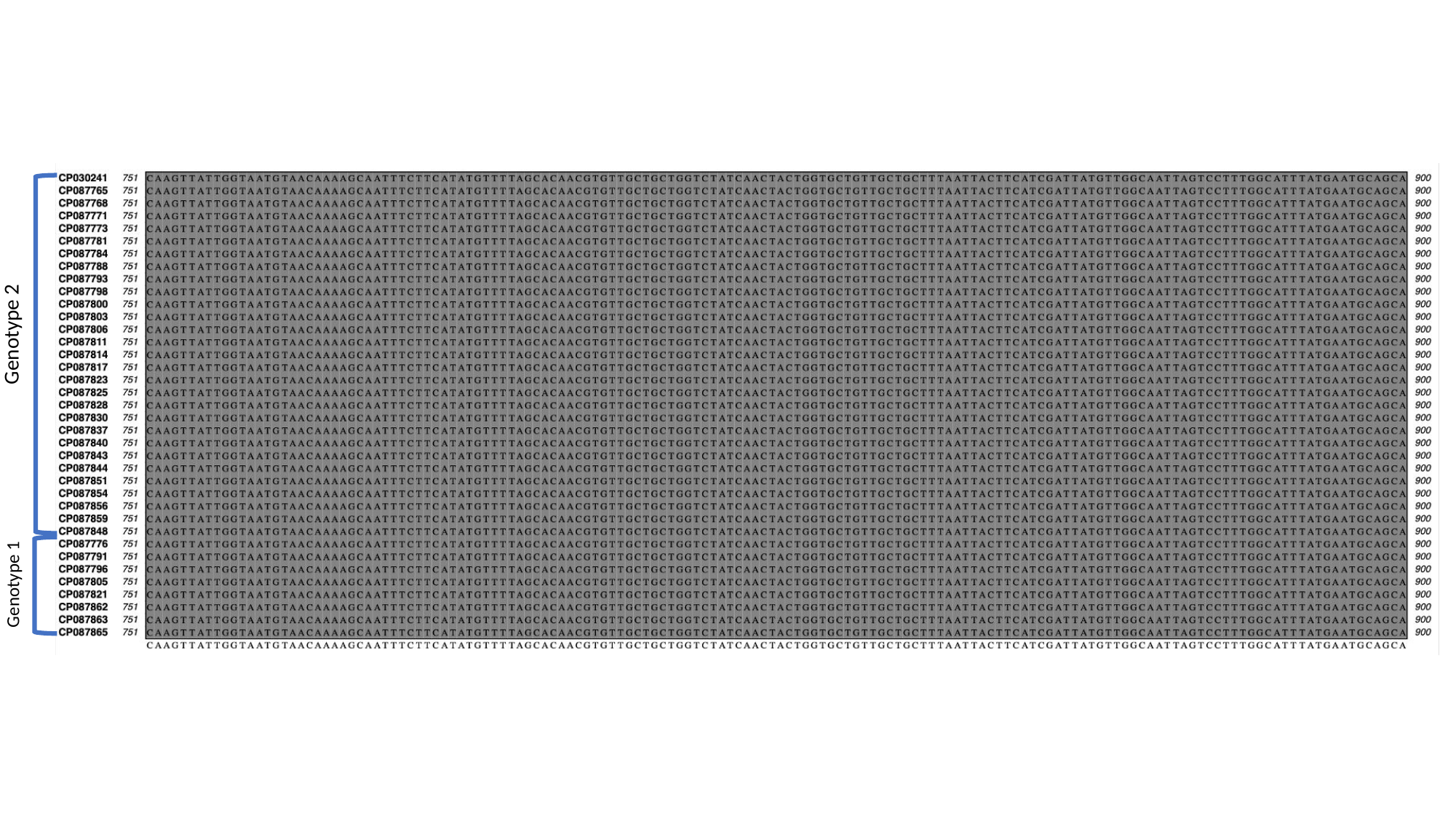

Genotype 2
Genotype 1

## Slide 7
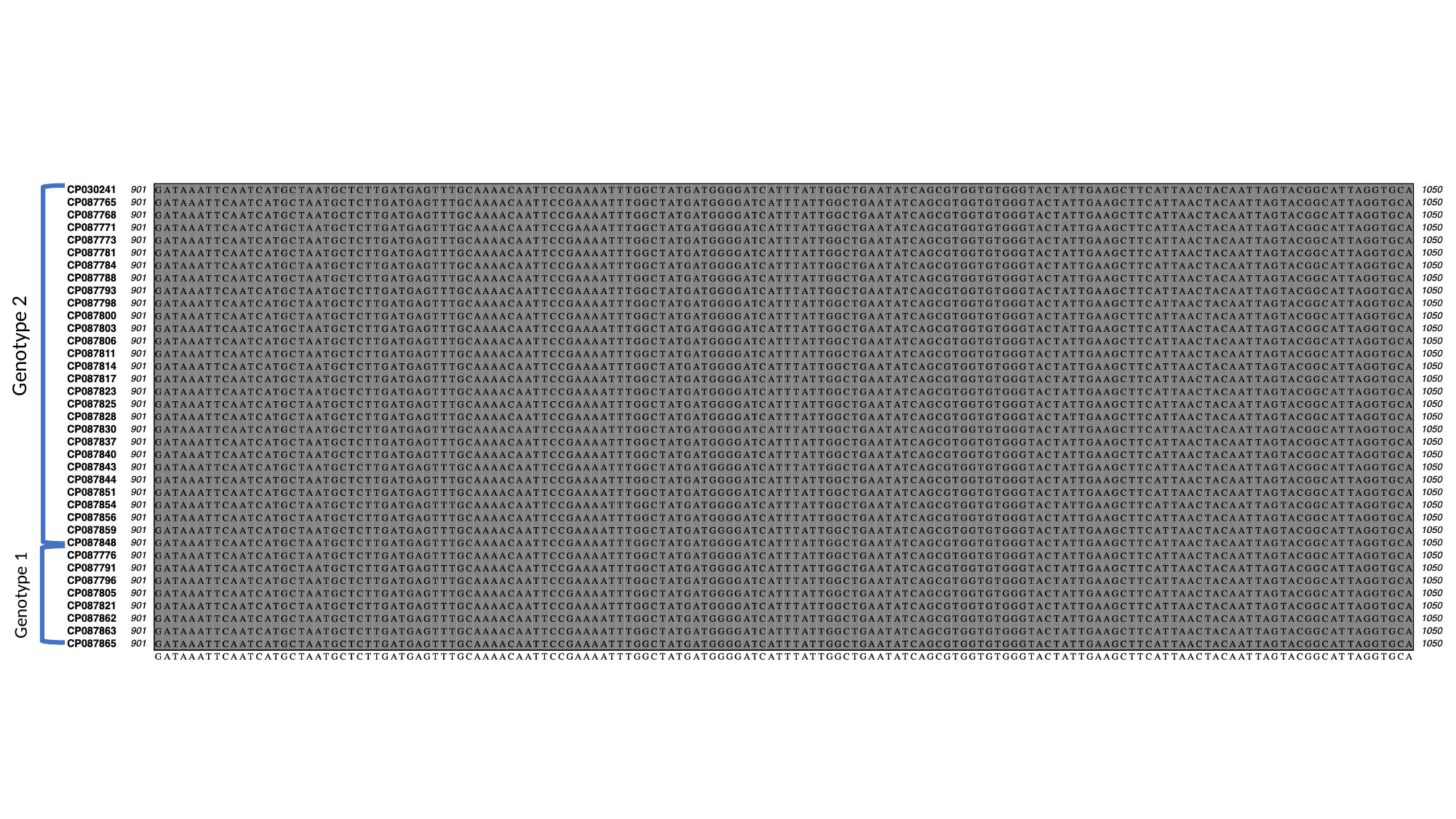

Genotype 2
Genotype 1

## Slide 8
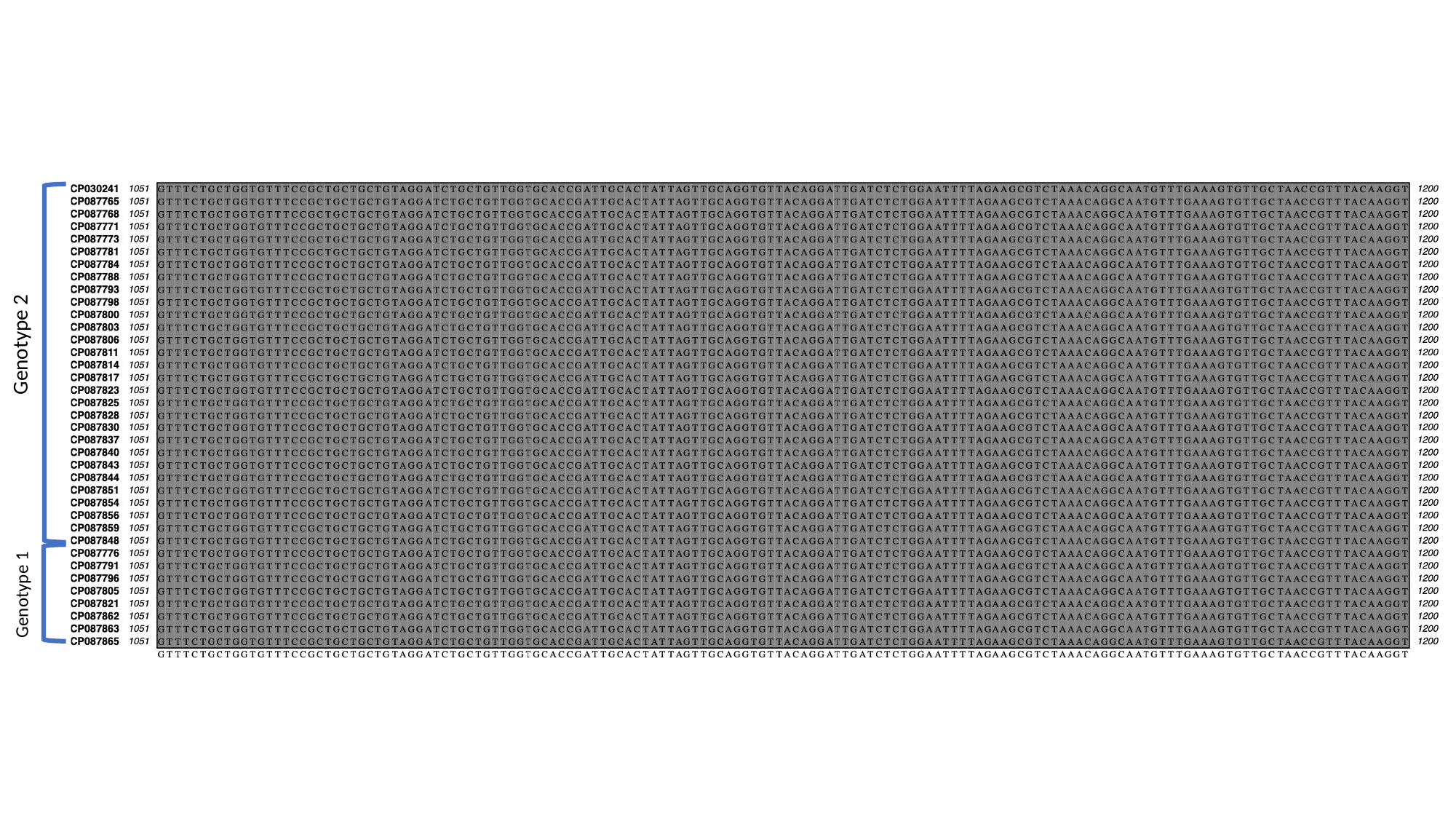

Genotype 2
Genotype 1

## Slide 9
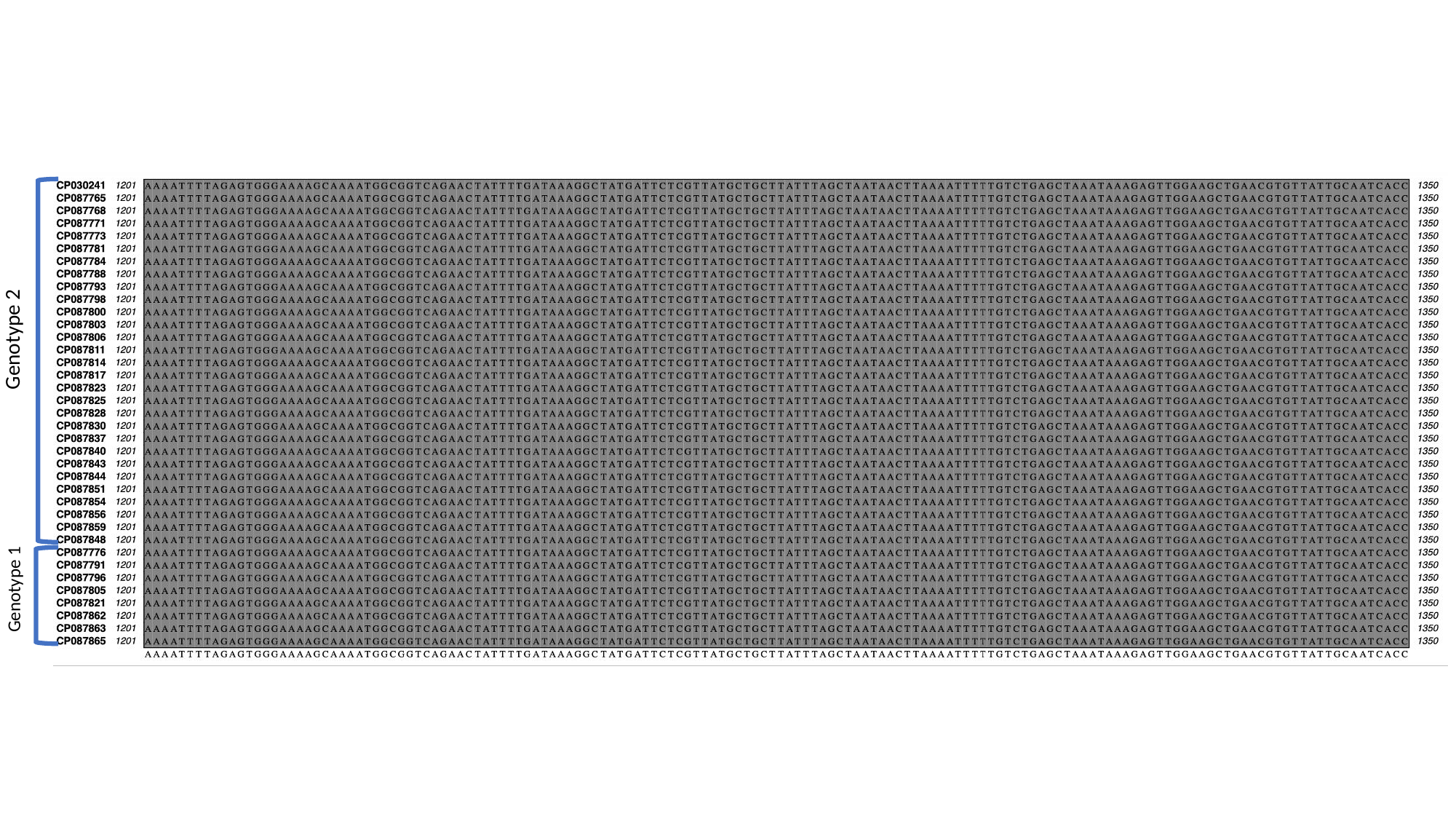

Genotype 2
Genotype 1

## Slide 10
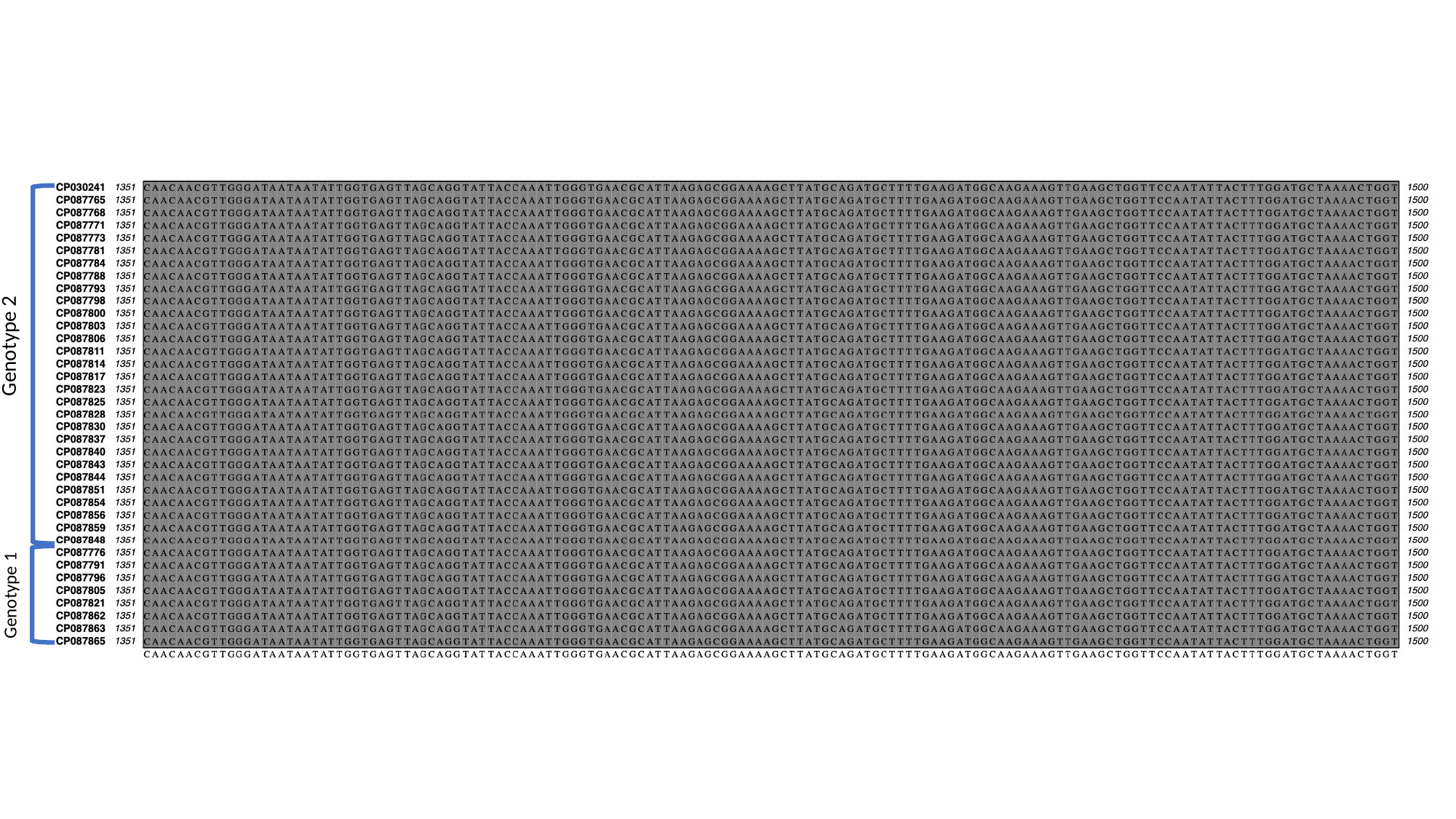

Genotype 2
Genotype 1

## Slide 11
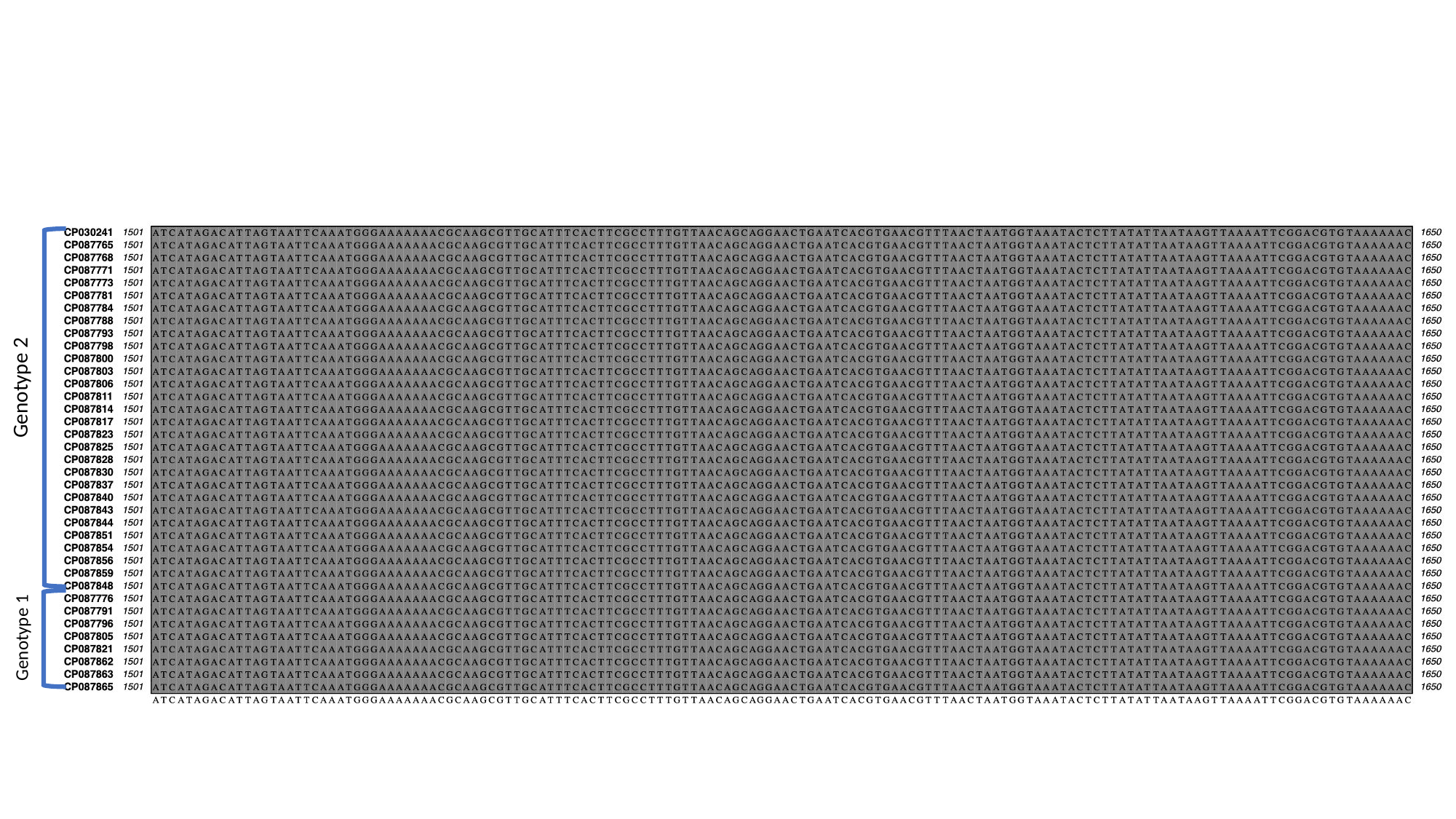

Genotype 2
Genotype 1

## Slide 12
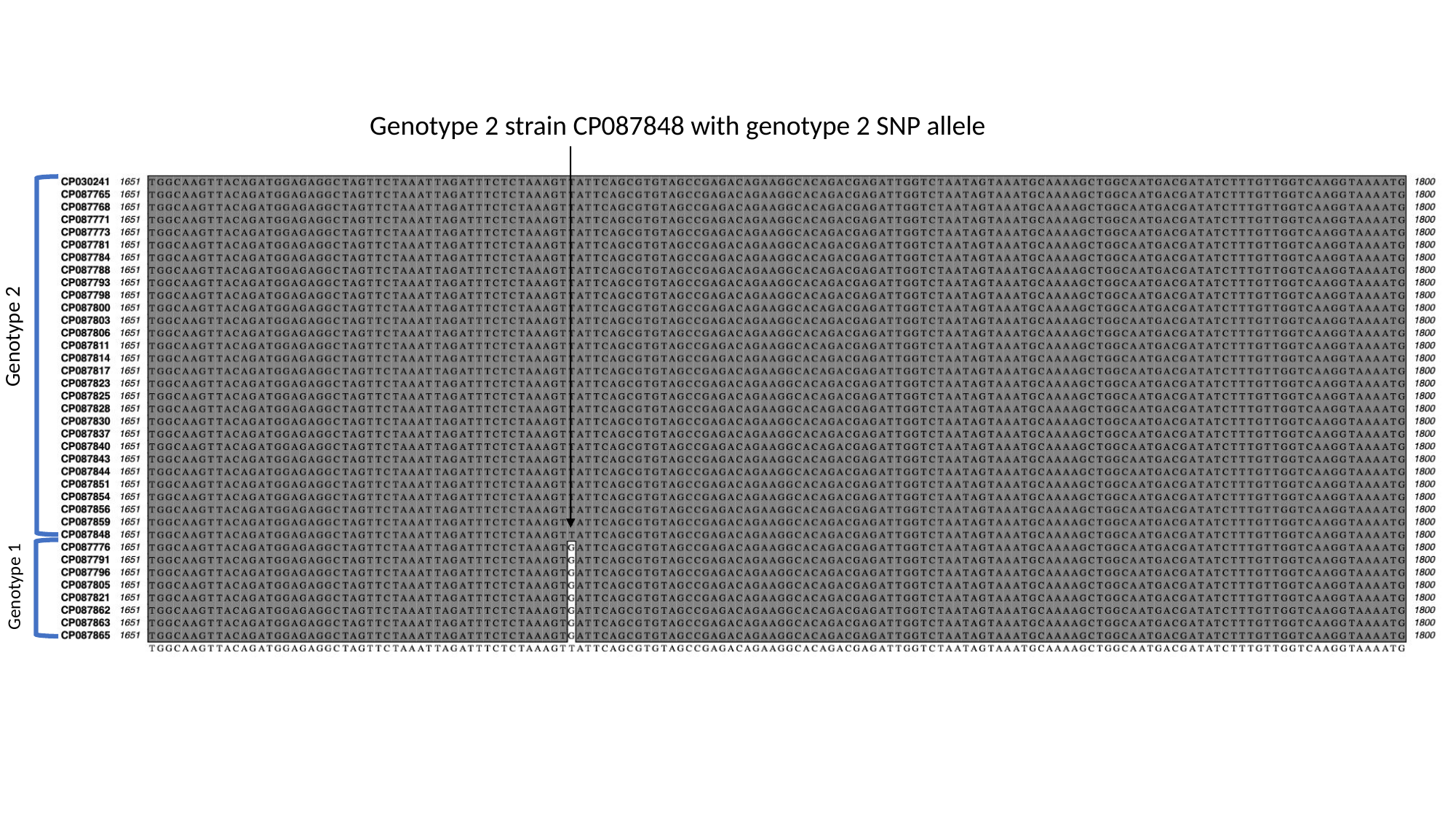

Genotype 2 strain CP087848 with genotype 2 SNP allele
Genotype 2
Genotype 1

## Slide 13
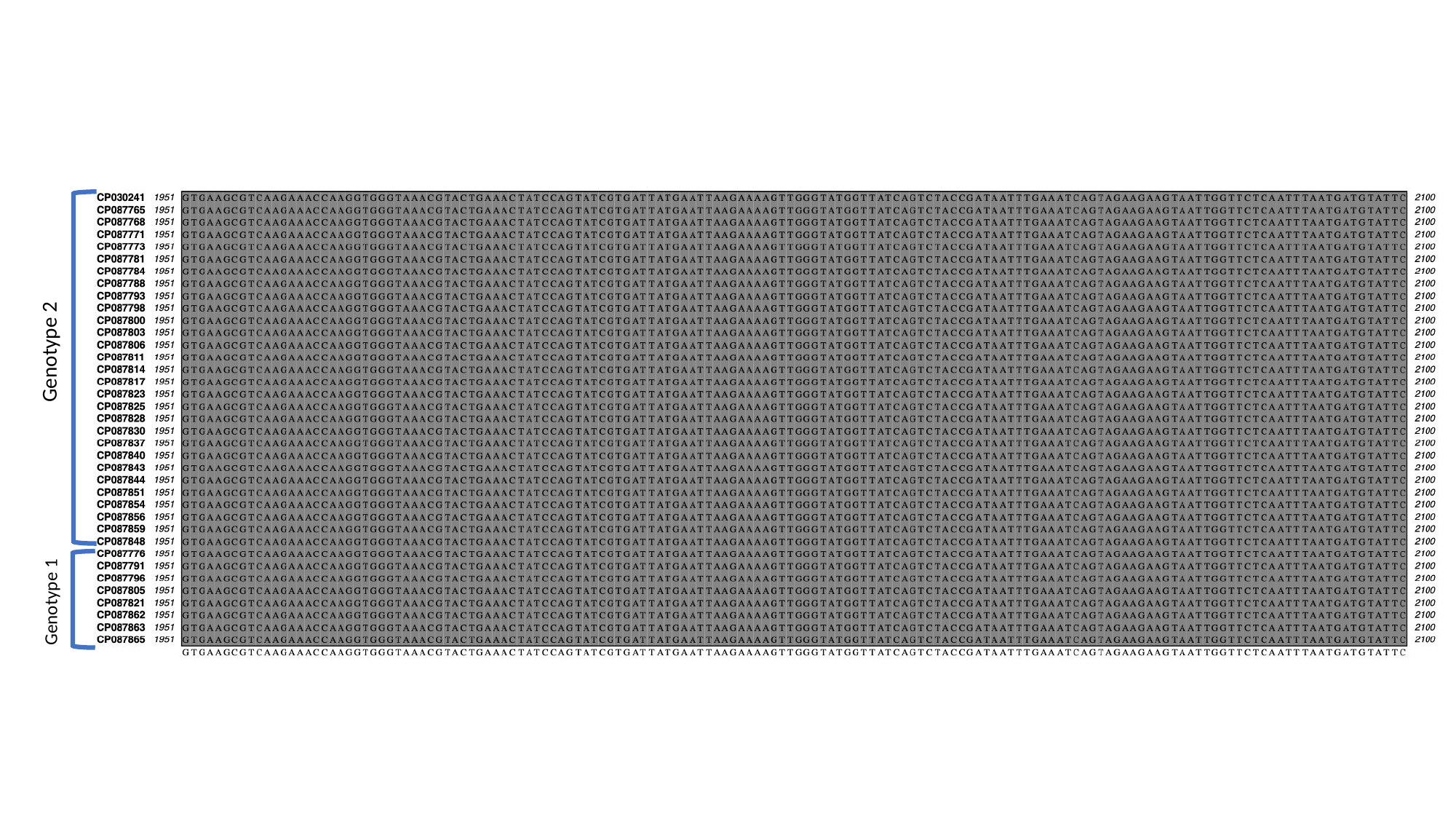

Genotype 2
Genotype 1

## Slide 14
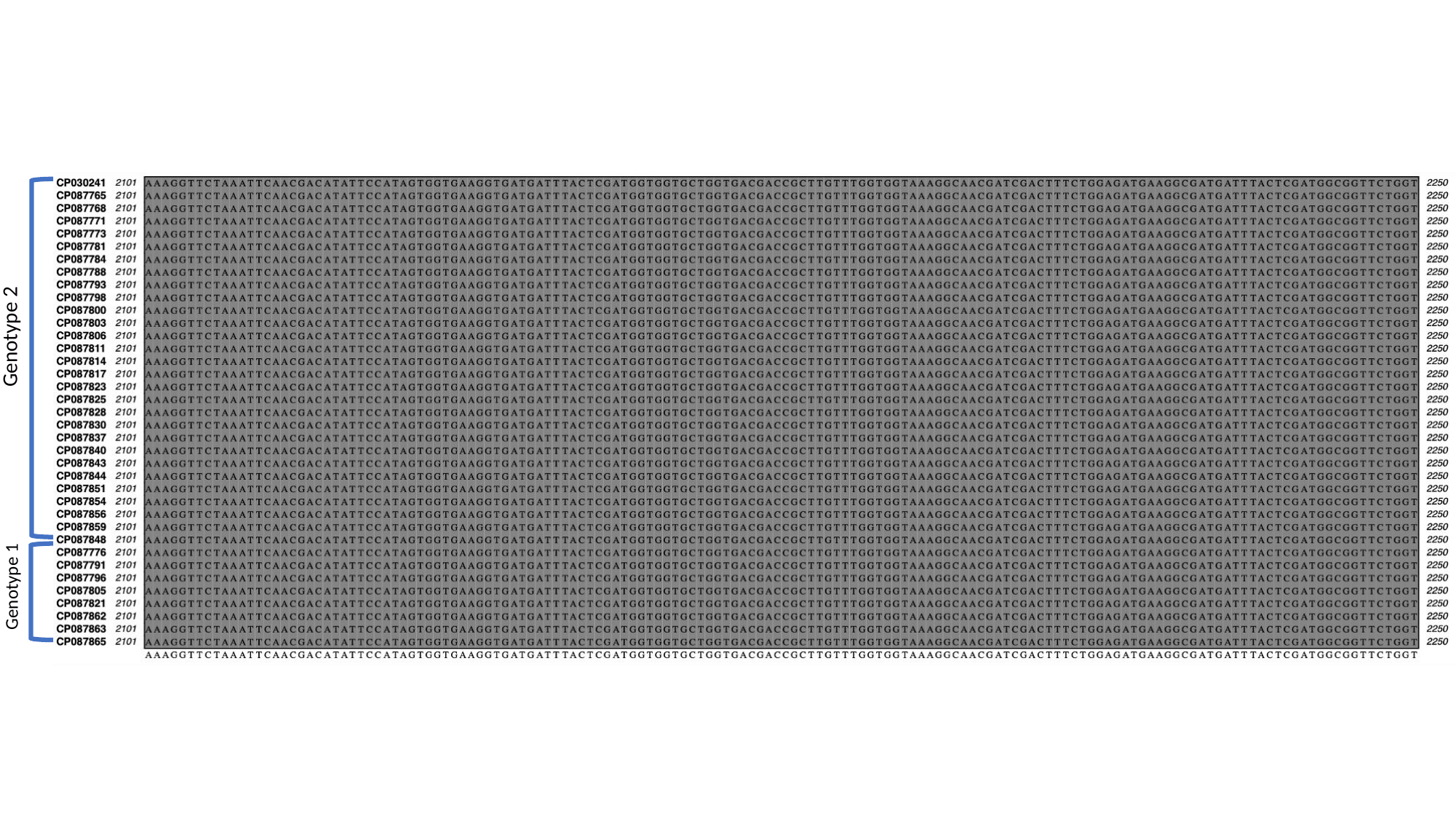

Genotype 2
Genotype 1

## Slide 15
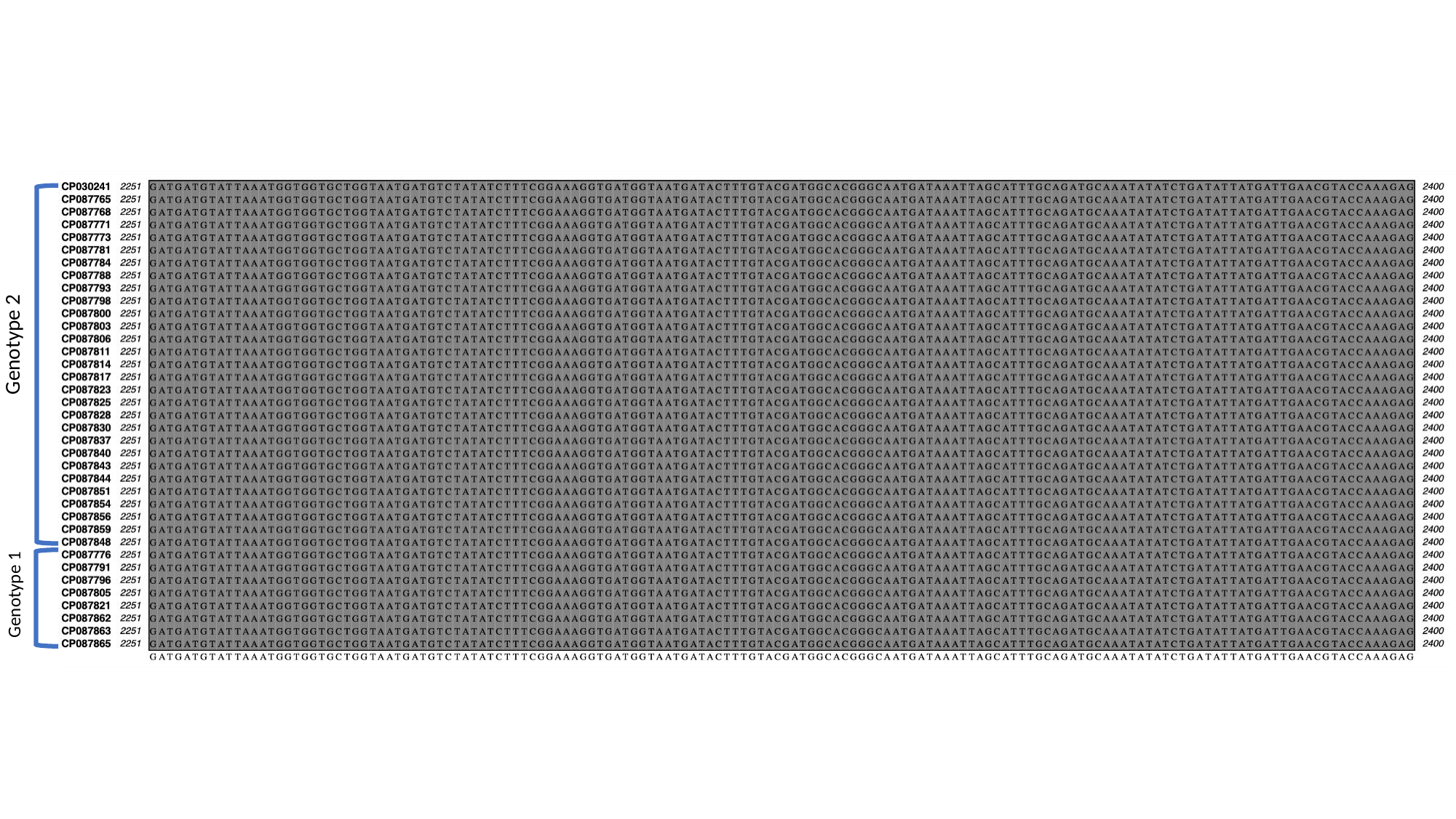

Genotype 2
Genotype 1

## Slide 16
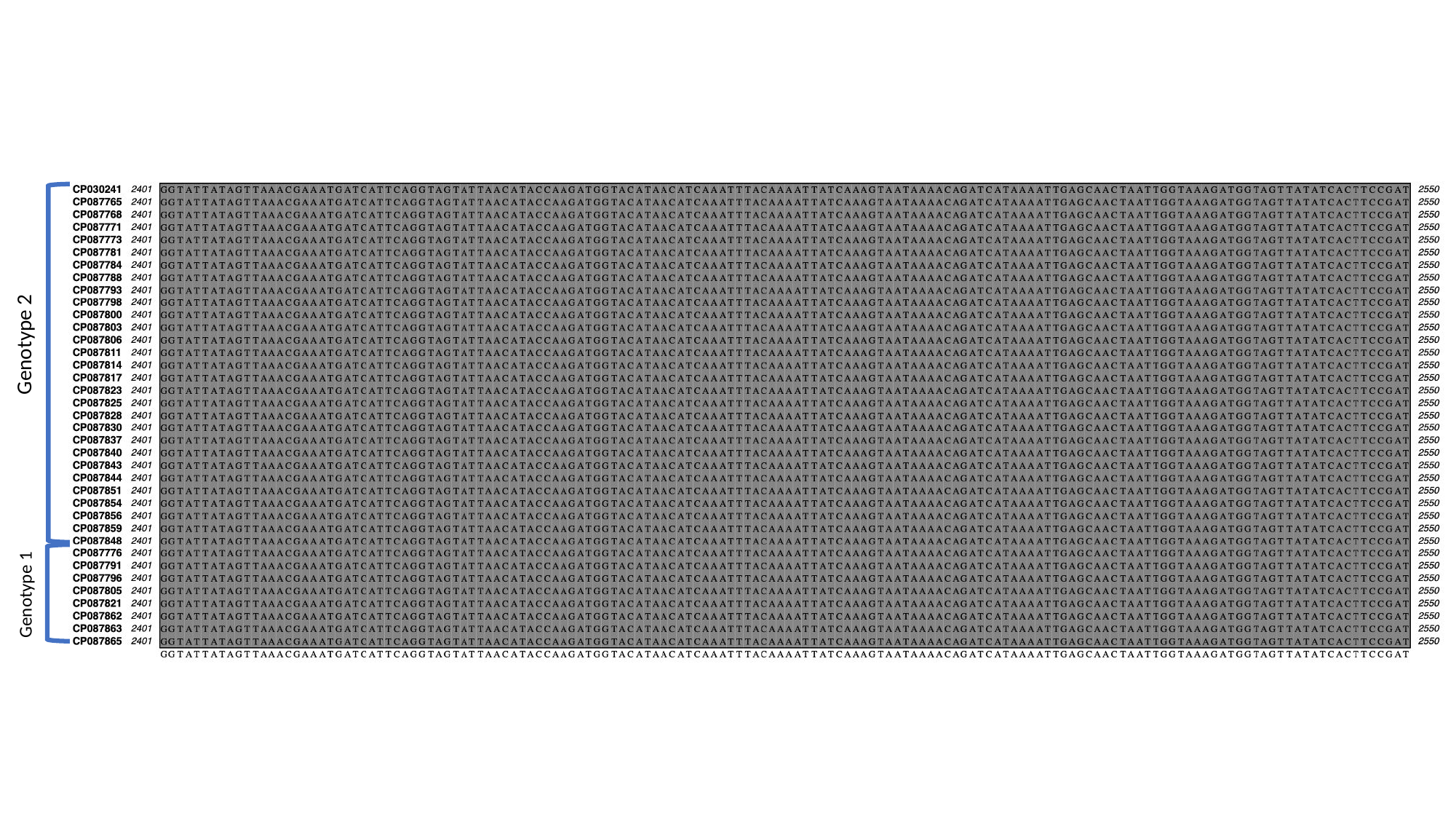

Genotype 2
Genotype 1

## Slide 17
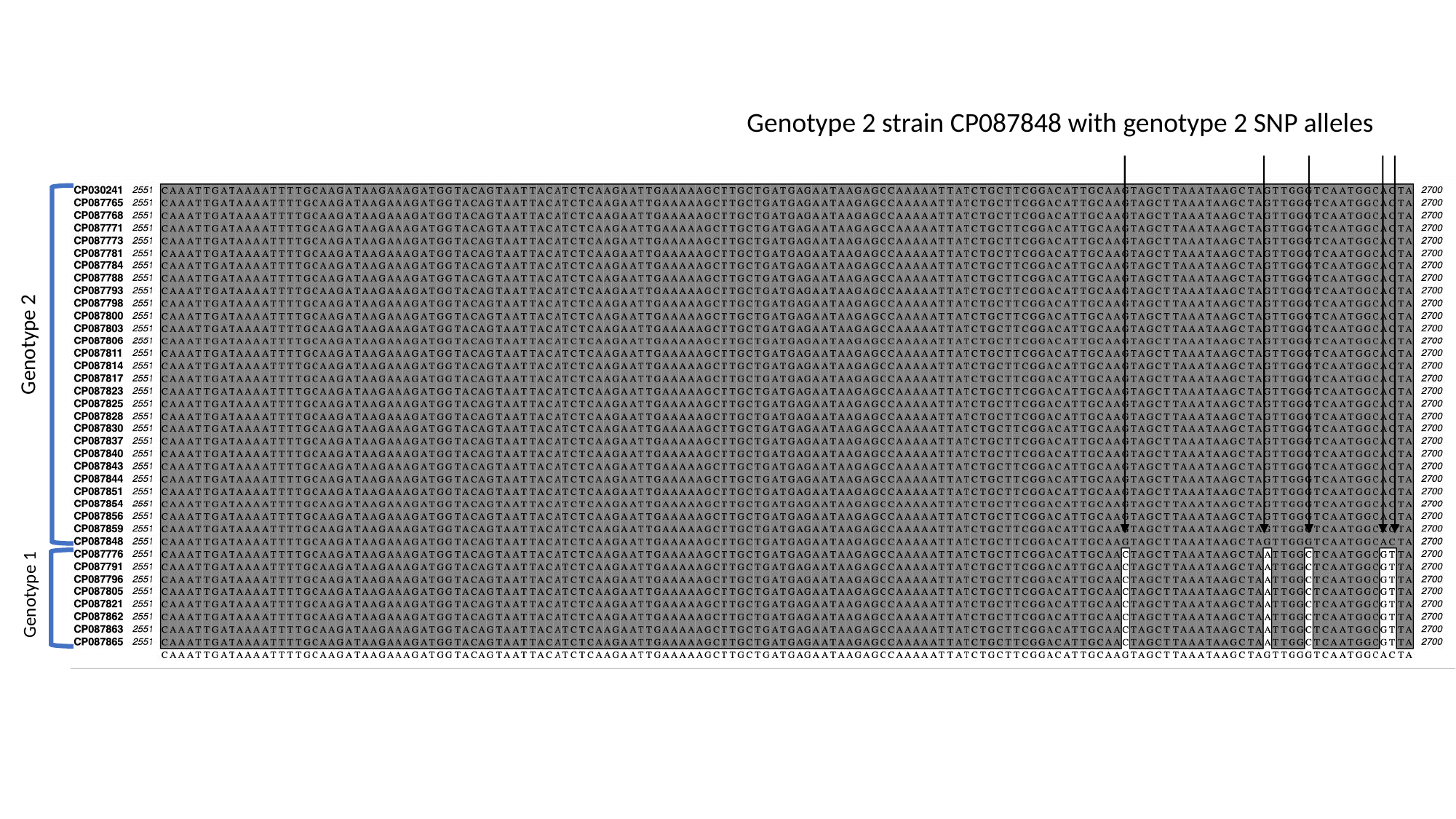

Genotype 2 strain CP087848 with genotype 2 SNP alleles
Genotype 2
Genotype 1

## Slide 18
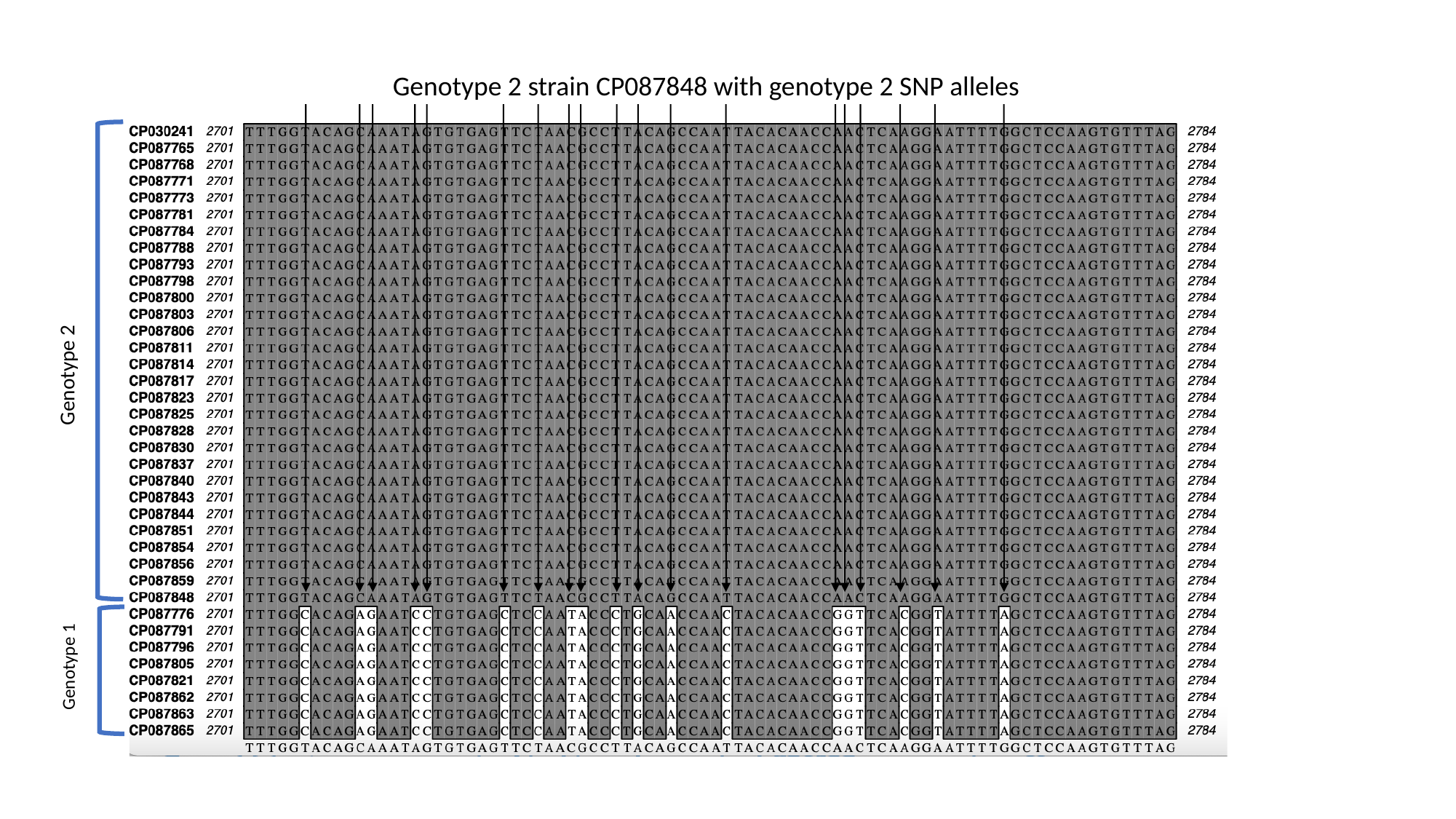

Genotype 2 strain CP087848 with genotype 2 SNP alleles
Genotype 2
Genotype 1
