# Supplementary material for: Whole genome sequencing of Moraxella bovis strains from North America reveals two genotypes with different genetic determinants
Source: BMC Microbiol. 2022 Oct 21;22:258. doi: 10.1186/s12866-022-02670-3 (PMC9585708; doi:10.1186/s12866-022-02670-3)
Supplement: Supplementary file 5 — Additional file 5. [file 12866_2022_2670_MOESM5_ESM.ppt]

## Slide 1
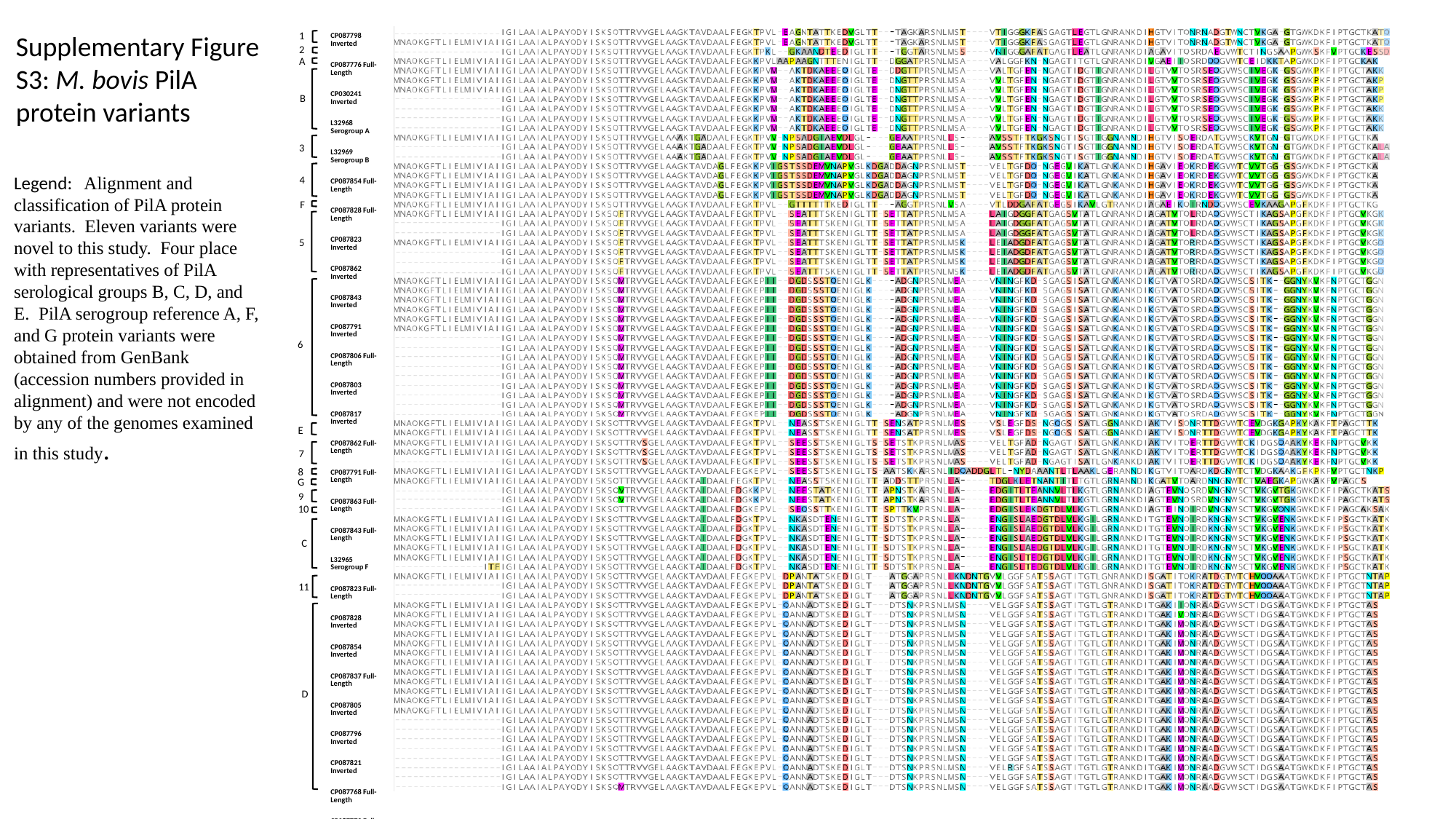

Supplementary Figure S3: M. bovis PilA protein variants
1
| CP087798 Inverted |
| --- |
| CP087776 Full-Length |
| CP030241 Inverted |
| L32968 Serogroup A |
| L32969 Serogroup B |
| CP087854 Full-Length |
| CP087828 Full-Length |
| CP087823 Inverted |
| CP087862 Inverted |
| CP087843 Inverted |
| CP087791 Inverted |
| CP087806 Full-Length |
| CP087803 Inverted |
| CP087817 Inverted |
| CP087862 Full-Length |
| CP087791 Full-Length |
| CP087863 Full-Length |
| CP087843 Full-Length |
| L32965 Serogroup F |
| CP087823 Full-Length |
| CP087828 Inverted |
| CP087854 Inverted |
| CP087837 Full-Length |
| CP087805 Inverted |
| CP087796 Inverted |
| CP087821 Inverted |
| CP087768 Full-Length |
| CP087773 Full-Length |
| CP087800 Full-Length |
| CP087859 Full-Length |
| CP087844 Full-Length |
| CP087771 Full-Length |
| CP087765 Inverted |
| CP087811 Inverted |
| CP087793 Inverted |
| CP087784 Inverted |
| CP087856 Inverted |
| CP087840 Inverted |
| CP087814 Inverted |
| CP087851 Inverted |
| CP087788 Inverted |
| CP087848 Full-Length |
| L32972 Serogroup E |
| CP087830 Full-Length |
| CP030241 Full-Length |
| CP087825 Inverted |
| CP087798 Full-Length |
| L32966 Serogroup G |
| CP087848 Inverted |
| CP087781 Inverted |
| CP087830 Inverted |
| CP087805 Full-Length |
| CP087821 Full-Length |
| CP087796 Full-Length |
| CP087825 Full-Length |
| CP087817 Full-Length |
| L32967 Serogroup C |
| CP087803 Full-Length |
| CP087863 Inverted |
| CP087806 Inverted |
| CP087851 Full-Length |
| CP087788 Full-Length |
| CP087814 Full-Length |
| CP087840 Full-Length |
| CP087856 Full-Length |
| CP087781 Full-Length |
| CP087784 Full-Length |
| CP087793 Full-Length |
| CP087811 Full-Length |
| CP087865 Full-Length |
| CP087765 Full-Length |
| L32971 Serogroup D |
| CP087771 Inverted |
| CP087844 Inverted |
| CP087800 Inverted |
| CP087773 Inverted |
| CP087768 Inverted |
| CP087776 Inverted |
| CP087837 Inverted |
| CP087859 Inverted |
2
A
B
3
Legend: Alignment and classification of PilA protein variants. Eleven variants were novel to this study. Four place with representatives of PilA serological groups B, C, D, and E. PilA serogroup reference A, F, and G protein variants were obtained from GenBank (accession numbers provided in alignment) and were not encoded by any of the genomes examined in this study.
4
F
5
6
E
7
8
G
9
10
C
11
D
